# Bacterial Homologs of Progestin and AdipoQ Receptors (PAQRs) Affect Membrane Energetics Homeostasis but Not Fluidity

**DOI:** 10.1128/jb.00583-21

**Published:** 2022-03-14

**Authors:** Maddison V. Melchionna, Jessica M. Gullett, Emmanuelle Bouveret, Him K. Shrestha, Paul E. Abraham, Robert L. Hettich, Gladys Alexandre

**Affiliations:** a Department of Biochemistry and Cellular and Molecular Biology, University of Tennessee, Knoxville, Tennessee, USA; b Institut Pasteur, UMR 2001 CNRS, Paris, France; c Bioscience Division, Oak Ridge National Laboratory, Oak Ridge, Tennessee, USA; d Genome Science and Technology, University of Tennessee, Knoxville, Tennessee, USA; NCBI, NLM, National Institutes of Health

**Keywords:** bacteria, fatty acid biosynthesis, membrane energetics, membrane potential, PAQRs

## Abstract

Membrane potential homeostasis is essential for cell survival. Defects in membrane potential lead to pleiotropic phenotypes, consistent with the central role of membrane energetics in cell physiology. Homologs of the progestin and AdipoQ receptors (PAQRs) are conserved in multiple phyla of *Bacteria* and *Eukarya*. In eukaryotes, PAQRs are proposed to modulate membrane fluidity and fatty acid (FA) metabolism. The role of bacterial homologs has not been elucidated. Here, we use Escherichia coli and Bacillus subtilis to show that bacterial PAQR homologs, which we name “TrhA,” have a role in membrane energetics homeostasis. Using transcriptional fusions, we show that E. coli TrhA (encoded by *yqfA*) is part of the unsaturated fatty acid biosynthesis regulon. Fatty acid analyses and physiological assays show that a lack of TrhA in both E. coli and B. subtilis (encoded by *yplQ*) provokes subtle but consistent changes in membrane fatty acid profiles that do not translate to control of membrane fluidity. Instead, membrane proteomics in E. coli suggested a disrupted energy metabolism and dysregulated membrane energetics in the mutant, though it grew similarly to its parent. These changes translated into a disturbed membrane potential in the mutant relative to its parent under various growth conditions. Similar dysregulation of membrane energetics was observed in a different E. coli strain and in the distantly related B. subtilis. Together, our findings are consistent with a role for TrhA in membrane energetics homeostasis, through a mechanism that remains to be elucidated.

**IMPORTANCE** Eukaryotic homologs of the progestin and AdipoQ receptor family (PAQR) have been shown to regulate membrane fluidity by affecting, through unknown mechanisms, unsaturated fatty acid (FA) metabolism. The bacterial homologs studied here mediate small and consistent changes in unsaturated FA metabolism that do not seem to impact membrane fluidity but, rather, alter membrane energetics homeostasis. Together, the findings here suggest that bacterial and eukaryotic PAQRs share functions in maintaining membrane homeostasis (fluidity in eukaryotes and energetics for bacteria with TrhA homologs).

## INTRODUCTION

Biological membranes are semipermeable barriers composed of proteins, which impart their biological functions, and lipids, which modulate biophysical properties (1). Maintaining membrane homeostasis is essential for cell survival. The lipid composition of membranes varies with cell type, organelle, and environmental conditions (2, 3). Membrane lipid composition, in particular its fatty acid moiety, imposes constraints on the physicochemical properties of the bilayer and controls membrane protein insertion, folding, and function (3, 4). Defects in membrane lipid homeostasis are associated with multiple cellular stresses and metabolic changes (5, 6). Organisms regulate the lipid composition of membranes using diverse mechanisms that target phospholipids and fatty acids (1, 7–9) to adjust membrane function to changes in the biophysical properties of the membrane bilayer (10).

The physicochemical properties of membranes are also essential for the maintenance of concentration and electrical gradients that comprise the membrane potential (Δψ) and power physiological work (11). The establishment of gradients across membranes directly results from their differential permeability to ions and charged molecules. Changes in Δψ have been best described in excitable cells such as neurons (12). However, recent experimental evidence indicates that most “nonexcitable” animal cells (12), as well as bacteria (13–15), have a dynamic Δψ. In bacteria, dynamic Δψ generates electrical signals that regulate intra- and interspecies communications in biofilms and serve as signals for mechanosensing (14, 16, 17).

Like eukaryotes, the bacterial Δψ is established through the activity of ion channels and transporters of monovalent/divalent cations and charged organic molecules (18–21). The bacterial transmembrane potential powers diverse physiological functions, including ATP synthesis, motility, pH homeostasis, electrical communication, resistance to antibiotics, cellular growth and division, and molecular transport (11). The transmembrane proton gradient (ΔpH) and the transmembrane electrical potential (ΔΨ) comprise the proton motive force (PMF), which plays an essential role in bacterial physiology (11). Homeostasis of the PMF is maintained by altering either the ΔpH or ΔΨ; if the ΔΨ is collapsed, bacteria compensate for this loss by increasing ΔpH (22). In both eukaryotes and bacteria, voltage-gated ion channels modulate their transport activity in response to changes in the electrical gradient across the membrane and are major regulators of the transmembrane electrochemical gradients (18, 23).

Here, we show that a membrane protein of unknown function in two Escherichia coli (encoded by *yqfA*) strains and the distantly related Bacillus subtilis (encoded by *yplQ*) modulates the ability of cells to maintain optimal (i.e., wild-type [WT]-like) membrane energetics, as reported by measuring alterations in dye fluorescence reflecting the changes in the membrane potential, which we refer to as membrane energetics homeostasis. We named this protein TrhA for “transmembrane homeostasis” protein A. TrhA is not related to voltage-gated channels or to transporters of ions, metals, or organic molecules but is related to the progestin and AdipoQ receptor (PAQR) family of proteins (24). PAQRs are conserved across multiple phyla of *Bacteria* and *Eukarya* (24). In eukaryotes, the PAQR superfamily includes three classes of membrane receptors (24). Class I PAQRs are related to the human adiponectin receptors (AdipoRs) and respond to adiponectin (25, 26). The PAQR homologs in humans and Caenorhabditis elegans function to maintain membrane fluidity and membrane fatty acid composition in response to conditions which rigidify the membrane (27–30). Class II PAQRs respond to progesterone (31–33). Class III PAQRs are phylogenetically closely related to bacterial TrhA/PAQR homologs (24). These proteins are proposed to function as hemolysin-III proteins because expression of recombinant bacterial homologs induces cytolysis of eukaryotic cells (34, 35). Results reported here implicate bacterial TrhA homologs in the maintenance of membrane energetics homeostasis, with membrane potential measurement as a reliable read-out.

## RESULTS

### The gene coding for TrhA is part of the E. coli FabR regulon.

**FIG 1:**
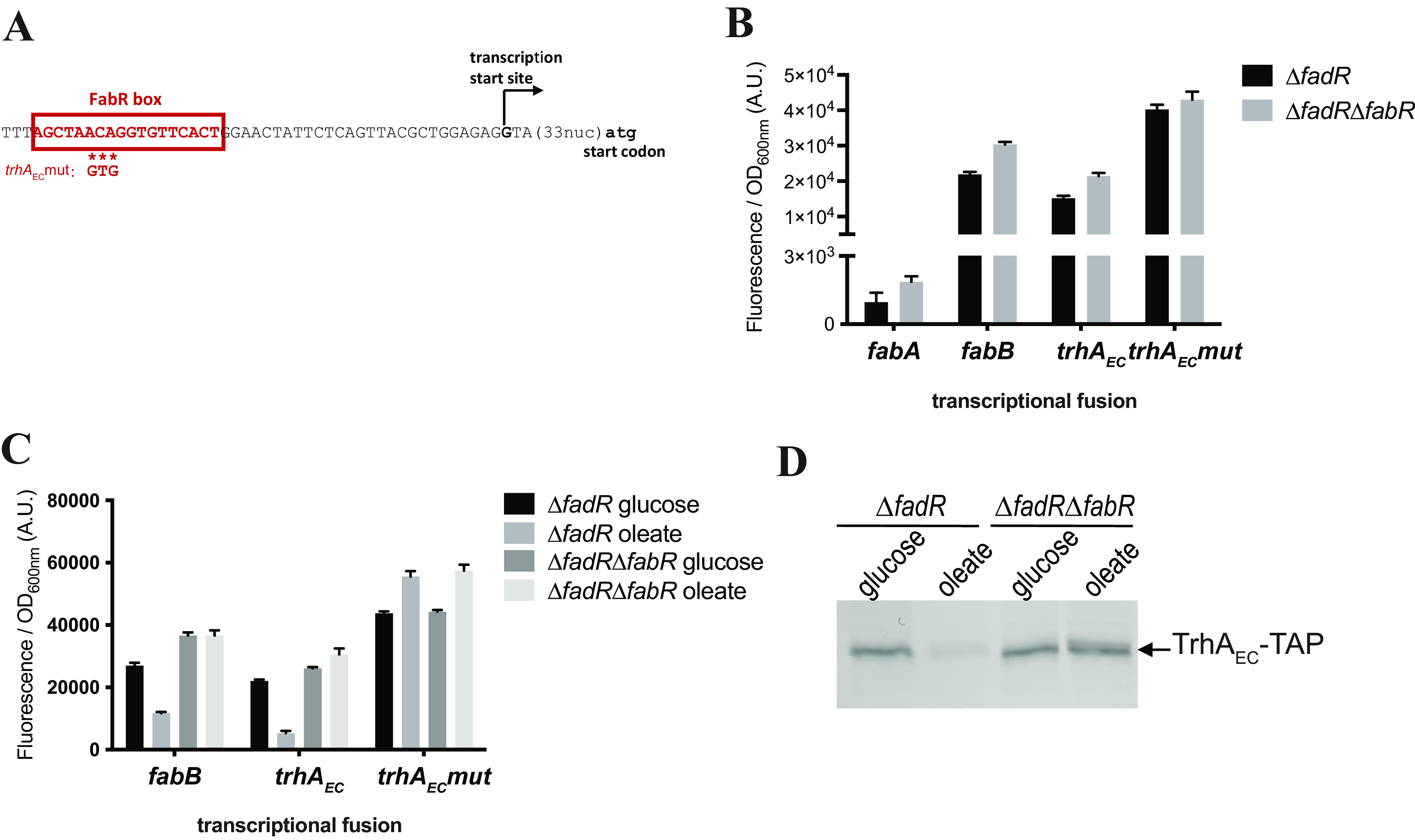
The gene coding for TrhA in E. coli is coregulated with genes of unsaturated fatty acid synthesis. (A) Sequence of the *trhA*_EC_ promoter. The transcription start site corresponds to the one identified in reference 38. The FabR box was identified in reference 36. The mutations introduced in the FabR box are indicated below the sequence. (B) Strains Δ*fadR* and Δ*fadR*Δ*fabR* were transformed by the plasmids containing the indicated transcriptional fusions. Relative fluorescence intensities were measured after overnight growth at 30°C in LB supplemented with kanamycin (see Materials and Methods). (C) Strains Δ*fadR* and Δ*fadR*Δ*fabR* were transformed by the plasmids containing the indicated transcriptional fusions. Relative fluorescence intensities were measured after overnight growth at 30°C in M9 minimal medium with 0.2% glucose or 0.2% oleate as the carbon source. (D) Strains Δ*fadR* and Δ*fadR*Δ*fabR* were transformed by the pUA-trhA-TAP plasmid. Cultures were performed at 37°C in M9 minimal medium with 0.2% glucose or 0.2% oleate as the carbon source. Total lysates were then analyzed by Western blotting using anti-TAP antibodies.

TrhA in E. coli (TrhA_EC_) is encoded by *yqfA.* In E. coli, several studies suggested that FabR, the repressor of unsaturated fatty acid synthesis genes, binds to the promoter of *yqfA* (*trhA_EC_*), in addition to the promoters of *fabA* and *fabB* (36, 37). The position of the FabR binding site relative to the transcription start site of *trhA_EC_* (38) is consistent with FabR acting as a repressor (Fig. 1A). However, the regulation of *trhA_EC_* by FabR *in vivo* suggested by global transcriptome analyses (39) was not confirmed in a subsequent study (36), maybe because the experiment in this later study was performed in the BW25113 strain reported to contain a mutation in *fabR* (39). We used transcriptional fusions with green fluorescent protein (GFP) (40) to compare the expression of *trhA_EC_* in the presence or absence of FabR in a Δ*fadR* genetic background to isolate the FabR regulation effects as previously described (36). The expression of *trhA_EC_* was upregulated in the Δ*fabR* strain as efficiently as for *fabB* (Fig. 1B). Mutations of three nucleotides in the FabR binding box in the promoter of *trhA_EC_* (*trhA_EC_* mut) increased *trhA_EC_* expression, while the *trhA_EC_* mut expression no longer increased in the Δ*fabR* mutant (Fig. 1B). Oleate, which promotes FabR repression, strongly repressed *trhA_EC_* expression, but not the *trhA_EC_* mut expression (Fig. 1C). This effect, dependent on the presence of FabR, was also observed by monitoring levels of TrhA_EC_-TAP protein (Fig. 1D). Taken together, these data confirm that *trhA_EC_* is regulated by FabR, suggesting that it may influence fatty acid membrane composition.

### The Δ*trhA_EC_* mutant derivative of E. coli has subtle changes in its fatty acid composition compared to E. coli WT.

**FIG 2:**
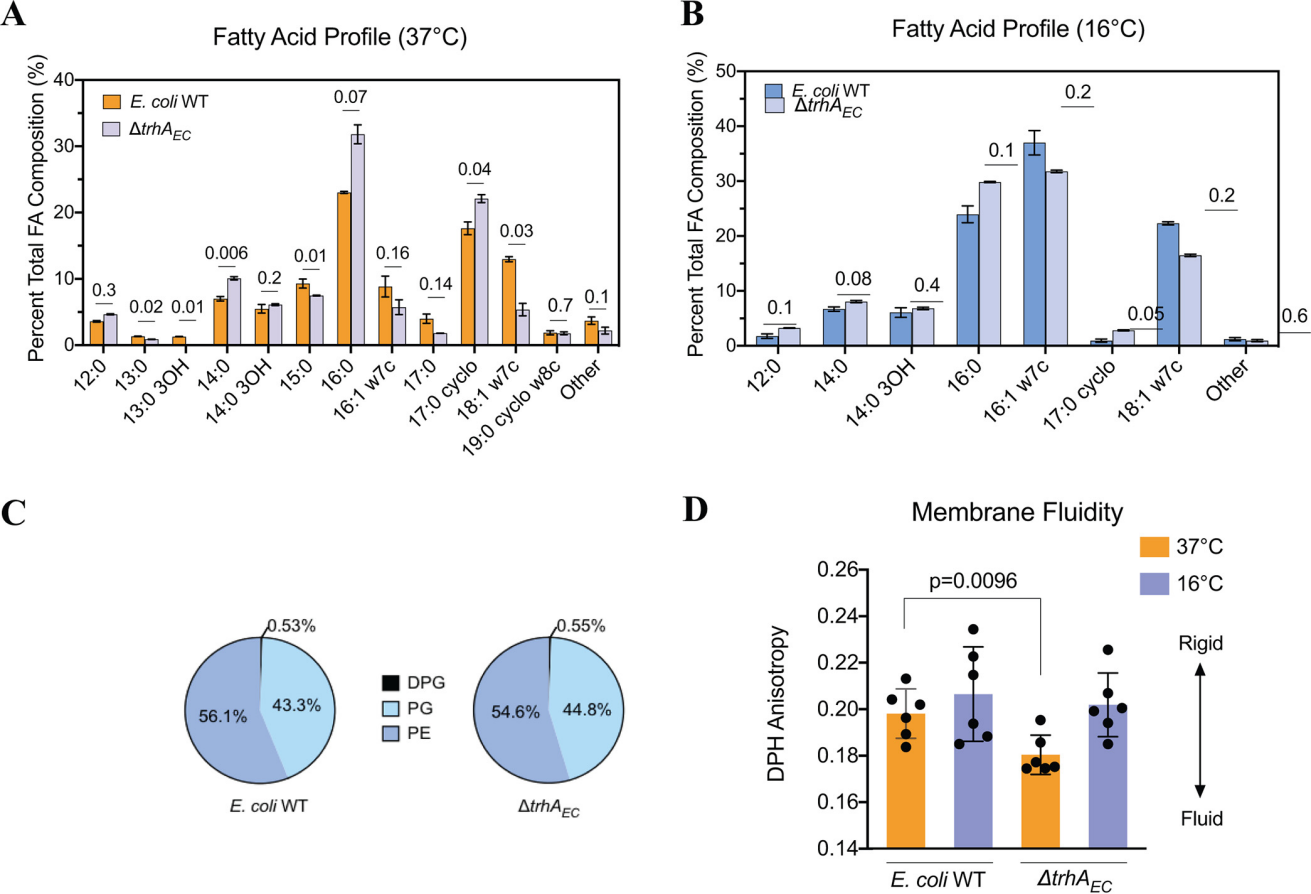
Control of membrane fluidity is not the primary role of TrhA_EC_. (A) Total fatty acid composition of E. coli WT and the Δ*trhA*_EC_ strain grown at 37°C. (B) Total fatty acid composition of E. coli WT and the Δ*trhA*_EC_ strain grown at 16°C. Error bars represent the standard deviation of two biological and two technical replicates. *P* values are listed above each set of bars. (C) Phospholipid headgroup analysis of E. coli WT and the Δ*trhA*_EC_ strain. The percent compositions of phosphatidylethanolamine (PE) lipids, diphosphatidylglycerol (PG) lipids, and diphosphatidylglycerol (DPG)/cardiolipin lipids are the averages from three biological replicates. (D) Membrane fluidity of E. coli WT and the Δ*trhA*_EC_ mutant. The membrane fluidity of whole cells was measured using DPH anisotropy, where greater values in anisotropy indicate a more rigid membrane. The data represent six biological replicates, where each point is the average of three technical replicates. Error bars represent the standard deviation.

The eukaryotic PAQRs characterized to date are implicated in FA metabolism or modification of phospholipids to adjust membrane fluidity (27–29, 41–45). Thus, we analyzed the FA composition of E. coli membranes from WT and strains lacking the TrhA homolog (Δ*trhA_EC_*) grown at physiological (37°C) and low (16°C) temperatures to challenge cells with membrane rigidity stress. When grown at 37°C, the Δ*trhA_EC_* strain produced fewer long, unsaturated C18:1w7c FAs and fewer C13:0 and C13:0 hydroxy FAs than the WT (Fig. 2A). It also produced more saturated C14:0 FAs and C17:0 cyclopropanes than the WT (Fig. 2A). At 16°C, the Δ*trhA_EC_* strain only increased its abundance of C17:0 cyclopropanes compared to the WT (Fig. 2B). Cells lacking TrhA_EC_ thus have subtle changes in membrane FA composition compared to the WT, but there were no significant differences in the proportions of phospholipid headgroups produced by either strain (Fig. 2C).

The Δ*trhA_EC_* mutant made adjustments to its FA profile similar to those of the WT (see Fig. S1A in the supplemental material). Both strains decreased their production of short, saturated FA and C17:0 cyclopropanes at 16°C compared to 37°C, and both increased production of long, unsaturated C16:1 w7c at 16°C (Fig. S1B). These findings indicate that TrhA_EC_ has small effects on FA composition, but it is not specifically implicated in a temperature-mediated membrane rigidity stress response.

### Membrane fluidity control is not the primary function of TrhA_EC_.

Next, we used 1,6-diphenyl 1,3,5-hexatriene (DPH) to compare the membrane fluidity of E. coli WT and the Δ*trhA_EC_* strain grown at 37°C and 16°C. The fluorescence anisotropy of DPH increases in ordered membranes and decreases in fluid membranes (46). For WT, the net membrane fluidity remained constant from 37°C to 16°C (Fig. 2D), consistent with E. coli maintaining fluidity across a range of growth temperatures (47). The Δ*trhA_EC_* mutant membrane was more fluid at 37°C than at 16°C and was comparable to that of E. coli WT at 16°C (Fig. 2D). These data are consistent with the subtle effects of a Δ*trhA_EC_* mutation on membrane FA composition at this temperature (Fig. 2B) and highlight an incongruency between the membrane fluidity and FA compositions of the Δ*trhA_EC_* strain at 37°C. The Δ*trhA_EC_* strain’s membrane is expected to be more rigid than that of the WT at 37°C, given the membrane FA profiles of the mutant (i.e., fewer long, unsaturated FAs and more saturated FAs than E. coli WT), but we found fluidity was increased (Fig. 2D). The primary function of TrhA_EC_ is thus unlikely to be membrane fluidity regulation.

### Membrane proteomics support a negligible effect on membrane fluidity.

**FIG 3:**
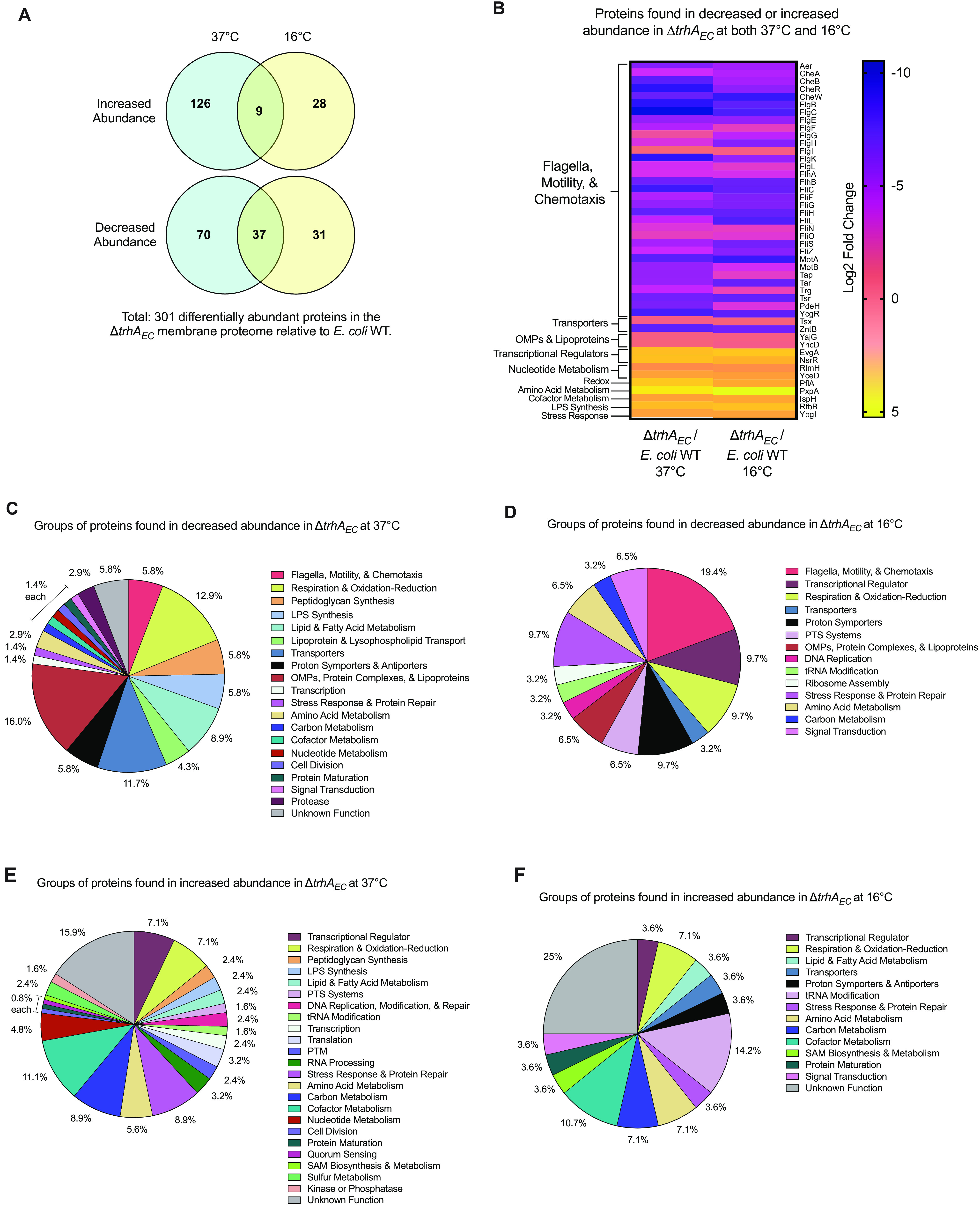
Membrane proteome of the Δ*trhA*_EC_ mutant reveals large changes in protein abundance, cellular physiology, and metabolism. (A) Venn diagram representing numbers of the differentially abundant proteins in the membrane proteome of Δ*trhA*_EC_ relative to E. coli WT. (B) Heat map of proteins that are found in decreased or increased abundance at both 37°C and 16°C in the membrane proteome of Δ*trhA*_EC_ relative to E. coli WT. The average log_2_ fold change of six biological replicates is represented. (C and D) Representation of proteins that are found in decreased abundance in the membrane proteome of ΔtrhA_EC_ relative to WT E. coli at (C) 37°C and (D) 16°C. Proteins are functionally classified into GO-terms. (E and F) Representation of proteins that are found in increased abundance in the membrane proteome of Δ*trhA*_EC_ relative to WT E. coli at (E) 37°C and (F) 16°C.

Next, we compared the membrane proteomes of E. coli WT and the Δ*trhA_EC_* strain, grown at 37°C and 16°C to identify membrane-specific function(s) associated with TrhA_EC_. The analysis captured soluble proteins that are overly abundant and/or which interact at the membrane. At both temperatures, Δ*trhA_EC_* had a membrane proteome with pleiotropic changes compared to the WT, suggesting a major role for TrhA_EC_ in cell physiology (Fig. 3). A total of 301 proteins were differentially abundant in the Δ*trhA_EC_* strain compared to E. coli WT and belonged to distinct functional groups based on Gene Ontology (GO-term) categories (Fig. 3 and Tables S1 and S2) (48).

Analysis of the Δ*trhA_EC_* mutant membrane proteome did not provide evidence for a prominent role in membrane fluidity. Compared to E. coli WT, the Δ*trhA_EC_* mutant produced lower abundances of FabA and FabF at 37°C (Table S1A) and slightly higher abundances of FabG at 37°C and FabH at 16°C (Table S1B). TesB, which is implicated in FA turnover, was more abundant in the Δ*trhA_EC_* mutant at 37°C compared to E. coli WT (Table S1B). These changes do not correlate with the net membrane fluidity increase of the mutant at 37°C; the increase in FA elongation enzyme FabG and decrease in unsaturated FA synthesis protein FabA are expected to result in a more rigid membrane for the mutant. Altered abundances of these proteins in the Δ*trhA_EC_* mutant were not common across the two temperatures, suggesting that other confounding factors affect FA composition and membrane fluidity in the mutant. No other proteins with known functions in FA metabolism that would suggest a role in membrane fluidity were differentially expressed. Outer membrane proteins (OMPs) and lipoproteins decreased in abundance in the Δ*trhA_EC_* proteome compared to the WT (Fig. 3C and D and Table S1A). Relative to the WT, proteins involved in cell surface structure and membrane remodeling increased and decreased in abundance, respectively, in the Δ*trhA_EC_* strain (Fig. 3C and D and Table S1A and B). These findings are consistent with TrhA_EC_ exerting pleiotropic effects on homeostasis of the membrane.

### E. coli strains lacking *trhA_EC_* are impaired in motility and membrane energetics.

**FIG 4:**
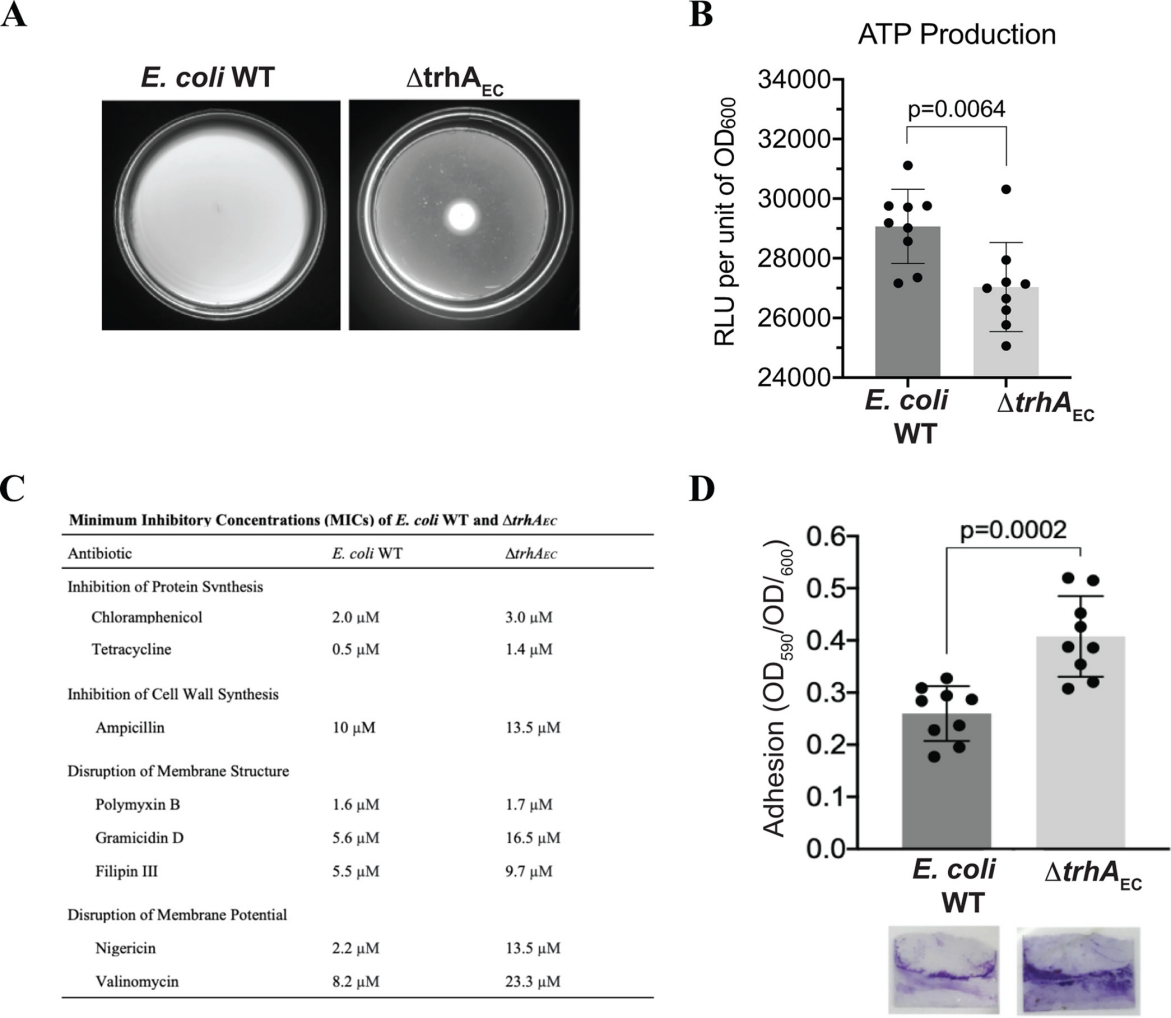
Physiology of the Δ*trhA_E_*_C_ mutant supports defective membrane energetics. (A) E. coli WT and Δ*trhA*_EC_ mutant cells were plated on the center of a soft agar (0.3%) plate. The plates were imaged after 18 h of incubation at 28°C. (B) The total ATP production of stationary-phase E. coli WT and Δ*trhA*_EC_ mutant cells was measured as luminescence relative light units (RLU). Data represent the ATP content from three biological replicates with three technical replicates. Error bars represent the standard deviation. (C) MICs of E. coli WT and the Δ*trhA*_EC_ strain for different antibiotics. MIC values represent the average concentration of antibiotic (in μM) that observably inhibited the growth of three to five biological replicates with three technical replicates each. (D) Biofilm production of E. coli WT and the Δ*trhA*_EC_ mutant. Cultures were grown overnight in 12-well plates containing a sterile microscope slide. Biofilms that adhered to the air-liquid interface of a coverslip were stained with crystal violet and quantified at OD_590_. The OD_590_ was normalized to cell growth as OD_600_. Points represent quantifications of biofilm produced from three biological replicates with three technical replicates. Error bars represent the standard deviation.

The most profound change in the Δ*trhA_EC_* membrane proteome relative to E. coli WT was the reduction in abundance of flagellar and chemotaxis proteins (Fig. 3B and Table S1B). Compared to E. coli WT we also observed by light microscopy that the Δ*trhA_EC_* mutant cells either did not swim or swam slowly in liquid media, suggesting that some flagella are present, and it did not chemotax in semisoft agar plates, consistent with impaired motility (Fig. 4A).

Membrane proteomics indicated that transporters, proton symporters, and antiporter proteins, most of which transport positively charged amino acids, divalent cations, and positively charged metals, were lower in abundance in the Δ*trhA_EC_* strain than in the WT (Fig. 3B to D and Table S1A). For example, substrate(s) denoted FeoB (Fe^2+^), HisQ (lysine, arginine, ornithine, and histidine), NikC (Ni^2+^), ZntA (Zn^2+^/Cd^2+^/Pb^2+^), and ZntB (Zn^2+^:H^+^) were reduced in abundance in the Δ*trhA_EC_* mutant relative to E. coli WT (Tables S1B and S2). Proteins involved in aerobic and anaerobic respiratory redox chains which pump protons, including dehydrogenases SdhD, NuoBJ, and HybA, and terminal reductases and oxidases NarGH, FrdD, and CyoA were less abundant in the mutant than in the WT (Fig. 3C and D and Table S1A). At 37°C, the mutant also reduced the abundance of three components of the ATP synthase complex (AtpDHG) (Table S1A). Consistent with these changes, the mutant produces less ATP than the WT (Fig. 4B). Proteomics thus suggest that the abundance of proteins that regulate or utilize gradients of charged molecules across the membrane, including protons, charged amino acids, metals such as iron, zinc, nickel (Table S1A), and ATP synthesis, are perturbed by the lack of TrhA. These changes are expected to affect membrane energetics.

### TrhA_EC_ in E. coli is required for optimal (WT-like) membrane potential.

**FIG 5:**
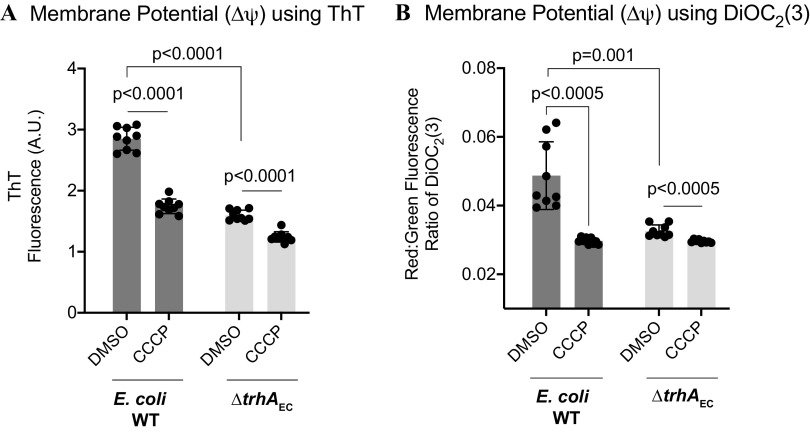
Cells lacking TrhA_EC_ have a depolarized membrane. (A and B) The membrane potential of E. coli WT and the Δ*trhA*_EC_ strain was measured using (A) ThT and (B) DiOC2(3) fluorescent reporters. DMSO treatments represent the resting membrane potential of cells. The membrane potential of each strain was collapsed when treated with CCCP, as indicated by decreased reporter fluorescence. The data represent fluorescence from three biological replicates with three technical replicates. Error bars represent the standard deviation.

To test the role of TrhA in membrane energetics, we used two fluorescent, hydrophobic, cationic reporters, DiOC_2_(3) and ThT, to compare the membrane potential (Δψ) of E. coli WT and the Δ*trhA_EC_* strain. DiOC_2_(3) and ThT fluoresce once accumulated within polarized cells (49), but their fluorescence decreases upon membrane depolarization (49). Depolarization using carbonyl cyanide m-chlorophenyl hydrazone (CCCP) caused DiOC_2_(3) and ThT fluorescence to decrease relative to the dimethyl sulfoxide (DMSO) controls in both WT and Δ*trhA_EC_* strains (Fig. 5A and B). However, the resting membrane potential (Δψ) of the Δ*trhA_EC_* mutant was significantly depolarized compared to that of the WT (Fig. 5A and B). While similar dyes have been used by others (e.g., 13–16), we note that fluorescent reporters for membrane potential are active within specific ranges (50). We expect a technical limitation in the ability to capture the absolute dynamic range of membrane potential.

**FIG 6:**
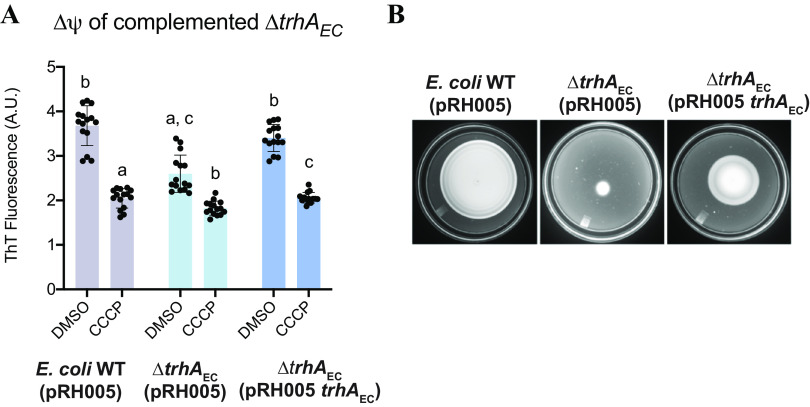
Functional complementation of the Δ*trhA*_EC_ strain by expressing WT *trhA*_EC_ using the pRH005 plasmid. (A) Membrane potentials of E. coli WT (pRH005), Δ*trhA*_EC_ (pRH005), and Δ*trhA*_EC_ (pRH005) were measured using ThT. Data represent fluorescence from five biological replicates with three technical replicates. Error bars represent the standard deviation. a, significantly different from E. coli WT (pRH005) DMSO (*P* < 0.05); b, significantly different from Δ*trhA*_EC_ (pRH005) DMSO (*P* < 0.05); c, significantly different from Δ*trhA*_EC_ (pRH005 *trhA*_EC_) DMSO (*P* < 0.05). (B) E. coli WT (pRH005), Δ*trhA*_EC_ (pRH005), and Δ*trhA*_EC_ (pRH005 *trhA*_EC_) cells were plated on the center of a soft agar (0.3%) plate. Plates were imaged after 18 h of incubation at 28°C.

Expressing parental *trhA_EC_* from a low-copy-number plasmid (51) in the Δ*trhA_EC_* strain (Δ*trhA_EC_* [pRH005 *trhA_EC_*]) restored the mutant Δψ to near-WT levels (Fig. 6A). This complemented Δ*trhA_EC_* mutant strain had a higher resting Δψ than the Δ*trhA_EC_* (pRH005) mutant that was not significantly different from that of the E. coli WT (pRH005) (Fig. 6A). The motility defect of the Δ*trhA_EC_* mutant was also partially complemented in this strain (Fig. 6B and Fig. S2A). Our attempts to perform functional complementation using inducers or different-copy-number plasmids (pTrc99a, pBAD33) failed to produce complementation (Fig. S2B and C). Poor transport of the inducers could be implicated here, given the altered membrane proteome and membrane energetics of Δ*trhA_EC_*. The inability to transport these inducers could contribute to the minimal expression of TrhA_EC_ from any plasmid or promoter. An alternative or additional possibility is that the cellular stoichiometry of TrhA_EC_ could also be a determining factor in its yet unknown function. Consistent with this notion, expressing pRH005 with *trhA*_EC_ in the parental E. coli WT strain, thereby increasing the abundance of TrhA_EC_, produces a dominant negative phenotype (Fig. S3A to C).

The effect of TrhA_EC_ on the mutant membrane potential compared to its parent but the lack of a measurable growth defect (Table S3) prompted us to analyze the sequenced genomes of the E. coli WT and Δ*trhA_EC_* strains. None of the few genotypic changes detected in the genome of the Δ*trhA_EC_* strain relative to the E. coli WT strain explained the effect of TrhA_EC_ on the membrane proteomes or energetics (Table S4). These mutations are either commonly found in laboratory strains of E. coli (*ins*B-5 and *ins*A-5) (52), are in a pseudogene (*stfE*), or have a predicted function in DNA recombination (*rmuC*). The only exception is a mutation in *pgk*, which encodes the glycolytic enzyme phosphoglycerate kinase (52). However, complementation of the Δ*trhA_EC_* strain using WT *pgk* in pRH005 did not restore motility to the mutant (Fig. S4A) or its membrane potential compared to the WT (Fig. S4B). These results are in agreement with functional (albeit partial) complementation of the *trhA* mutation and the dominant negative phenotype upon overexpression of *trhA* described above.

### Modified metabolism and multiple stresses are associated with dysregulated membrane energetics.

Consistent with altered membrane energetics, metabolic changes were evidenced in the mutant proteome relative to the WT (Fig. 3B to F and Tables S1 and S2). The Δ*trhA_EC_* mutant increased abundances of proteins which synthesize cofactors (cofactor annotated)—CoaA (coenzyme A), PanC (pantothenate), IspAE (isoprenoids), LipA (fatty acid anion lipoate), UbiC (ubiquinone), NadE (NAD^+^), HemHL (heme), MoeA (molybdenum), RibC (riboflavin), ThiL (thiamine), and EntC (enterobactin) (Fig. 3B, E, and F and Table S1B). Enzymes involved in glycolysis, the glyoxylate shunt pathway, and the production of glyoxylate, increased in the mutant relative to the WT. Changes in abundance of proteins were implicated in amino acid and nucleotide metabolism (Fig. 3B, E, and F and Tables S1B and S2). DNA replication and modification (Fig. 3C to F and Tables S1B and S2), transcription, and translation (Fig. 3E and F and Tables S1B and S2) were affected in the mutant relative to the wild type. These changes suggest that the mutant’s metabolism is dedicated to conservation of carbon, replenishment of metabolic intermediates, turnover pool of cofactors, and remodeling of information transfer processes.

Stress resistance responses were increased in the mutant compared to E. coli WT. The abundance of proteins with function in DNA damage repair and iron-sulfur cluster repair as well as glutaredoxin systems which help maintain oxidative states of small redox-sensitive thiol sensor proteins (53) increased in the *ΔtrhA_EC_* mutant proteome (Fig. 3E and F and Table S1B), suggestive of upregulation of oxidative stress pathways. Reduction in the abundance of Cyo and Nuo proton-pumping complexes in the Δ*trhA_EC_* mutant compared to the WT could suggest mild alkaline stress (Fig. 3B to D and Table S1A), as E. coli reduces these complexes when grown at alkaline pH (54). The abundance of several transcriptional regulators important for acid and osmotic stress responses increased in the Δ*trhA_EC_* strain compared to E. coli WT (Fig. 3E and F and Table S1B). The mutant had increased abundances of EvgA, which mediates acid resistance, osmotic adaptation, and drug resistance and represses motility (55), as well as OmpR, which plays a central role in acid stress response and is involved in osmoregulation (56) (Table S1B). Additional evidence of the mutant’s increased acid stress responses included increased abundance of glutamic acid decarboxylase regulator GadW and hydrolase PxpA, which consumes ATP to form l-glutamate (52) (Table S2). The abundance of enzymes that break down amino acids to release ammonium ions, which would acidify the cytoplasm, decreased in the Δ*trhA_EC_* mutant compared to the WT (Fig. 3C and D and Table S1A). Growth of the Δ*trhA_EC_* mutant, despite a depolarized membrane potential relative to the WT, is thus likely at the expense of reduced metabolism, motility, and expression of acid, alkaline, osmotic, and/or oxidative stress responses. These data also support that changes in the membrane potential, as reported here with fluorescent dyes, are relevant read-outs to further probe the role of TrhA.

### The Δ*trhA*_EC_ mutant was less affected than the WT under stressful growth conditions.

**FIG 7:**
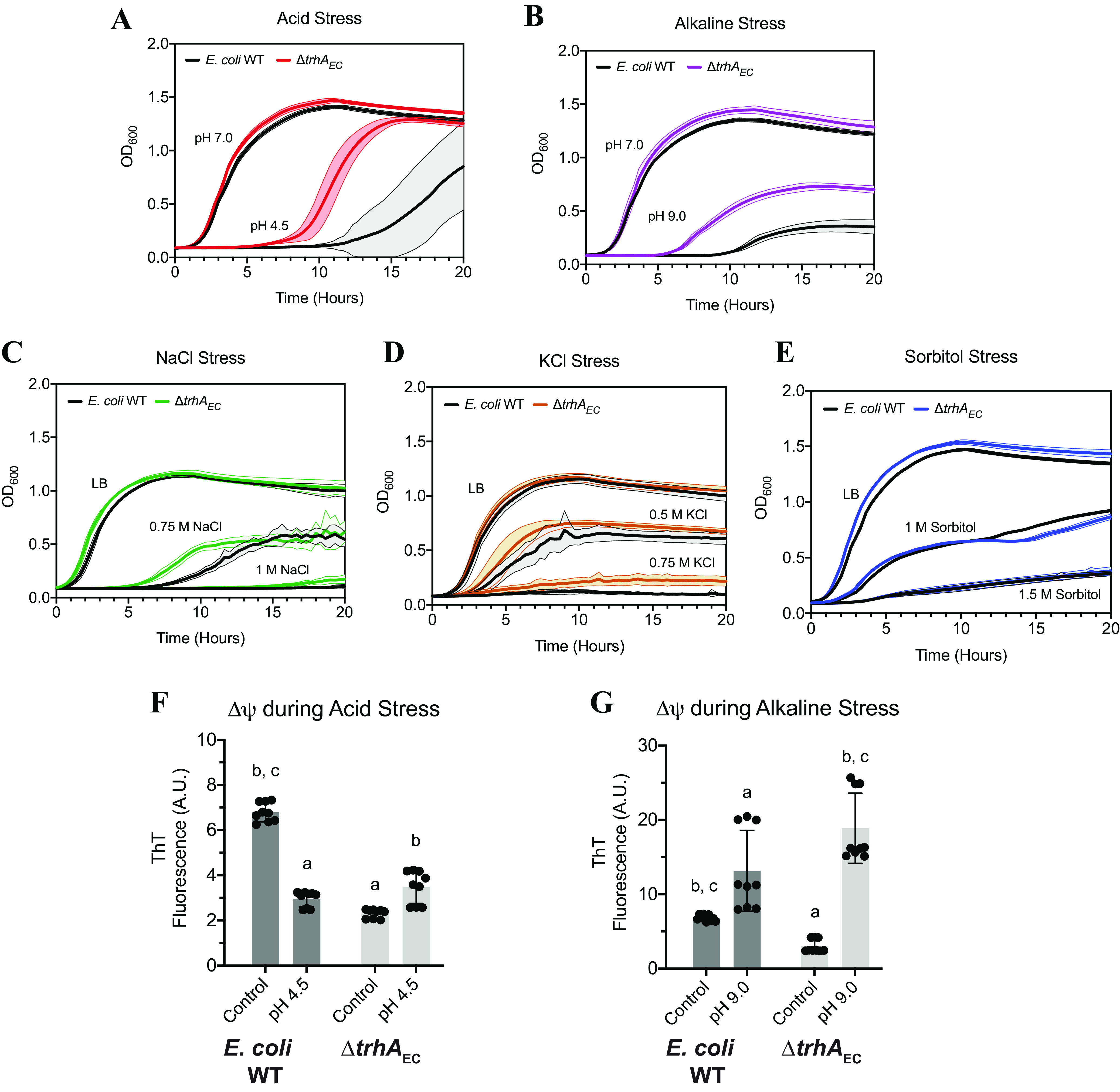
The Δ*trhA*_EC_ mutant is preadapted to external stressors that perturb charge or ion gradients across the membrane. (A to E) E. coli WT and the Δ*trhA*_EC_ mutant cells were grown to the logarithmic phase at 37°C in LB medium at pH 7.0 and then shifted to (A) acid stress at pH 4.5, (B) alkaline stress at pH 9.0, (C) NaCl stress at 0.75 M or 1 M NaCl, (D) KCl stress at 0.5 M or 0.75 M KCl, or (E) sorbitol stress at 1 M or 1.5 M sorbitol. All growth curves are representative of the average and standard deviation of three biological replicates with three technical replicates. (F and G) The membrane potential of E. coli WT and the Δ*trhA*_EC_ mutant was measured using (F) ThT during acid stress at pH 4.5 or (G) alkaline stress at pH 9.0. All measurements represent the membrane potential measurements from three biological and three technical replicates. Error bars represent the standard deviation. a, significantly different from E. coli WT control (*P* < 0.05); b, significantly different from Δt*rhA*_EC_ control (*P* < 0.05); c, significantly different from E. coli WT stress treatment (pH 4.5 or pH 9.0) (*P* < 0.05).

To test the hypotheses generated by the proteomics data described above, we compared the abilities of E. coli WT and the Δ*trhA_EC_* mutant to grow under acid, alkaline, and osmotic stress. We did not test oxidative stress, since it is associated with acid or osmotic stresses (54). When exposed to acid stress, the Δ*trhA_EC_* mutant resumed logarithmic growth 6 h postshift at pH 4.5, which was shorter than the 10-h delay for E. coli WT (Fig. 7A). The Δ*trhA_EC_* mutant grew faster than the WT at pH 4.5, and it reached a maximal optical density close to that of both the WT and Δ*trhA_EC_* at pH 7.0 (Fig. 7A). The fast growth of the mutant at pH 4.5 correlated with an increased Δψ relative to its Δψ at pH 7.0 (Fig. 7F). This pattern was opposite to the depolarization of the E. coli WT Δψ at pH 4.5 compared to pH 7.0 (Fig. 7F). When grown under alkaline stress (pH 9.0), the Δ*trhA_EC_* mutant had a shorter lag phase and grew faster and to a higher cell density than the WT (Fig. 7B). The maximal cell density of the mutant at alkaline pH remained low compared to growth at pH 7.0, indicating that the mutant still experienced stress, albeit not as severely as that WT (Fig. 7B). At pH 9.0, the Δψ of WT and Δ*trhA_EC_* strains became hyperpolarized (Fig. 7G), but the increase in Δψ was greater for the Δ*trhA_EC_* mutant strain than for the WT, consistent with the mutant’s faster growth under these conditions (Fig. 7G). The mutant lacking TrhA is not able to adjust its membrane potential to levels similar to those of the WT when challenged with conditions causing proton gradient stress. The observations that the mutant’s membrane potential is hyperpolarized under both acid and alkaline stress compared to the WT under similar conditions further suggest the that mutant is impaired in its ability to adjust membrane potential with changing conditions.

Next, we manipulated charge gradients through osmotic stress. In the presence of 0.75 M NaCl, the Δ*trhA_EC_* strain initiated growth roughly 5.5 h post-exposure to the stress, while E. coli WT began to grow after about 7 h (Fig. 7C). With 1.0 M NaCl, the mutant showed minimal slow growth after approximately 17 h post-stress exposure, while the E. coli WT did not grow (Fig. 7C). These differences are consistent with the mutant being preacclimated to osmotic stress suggested by proteomics. High concentrations of NaCl produced a similar growth defect in the E. coli WT and the Δ*trhA_EC_* mutant, with both strains reaching equivalent low maximum densities under these conditions, consistent with the two strains experiencing similar osmotic stress (Fig. 7C). Similar small growth advantages of the mutant relative to the WT were observed using KCl (Fig. 7D). There was no such preacclimation or growth advantage for the mutant relative to the WT when the uncharged sorbitol was used to provoke osmotic stress (Fig. 7E). These findings suggest the preacclimation of the mutant is specific to changes in the concentrations of protons or salt in the cells’ environment.

### Additional physiological changes of the Δ*trhAEC* mutant support a defective Δψ.

Cells with a perturbed membrane potential have pleiotropic defects, consistent with the central role of membrane potential in cellular physiology (57). Susceptibility to some antibiotics is associated with a perturbed membrane potential (57). The Δ*trhA_EC_* mutant was more resistant to antibiotics representing different mechanisms of action—protein (chloramphenicol and tetracycline) or cell wall (ampicillin) synthesis, membrane permeability (gramicidin D and filipin III), and membrane potential (nigericin and valinomycin) (Fig. 4C). One exception was polymyxin B, for which we did not detect any difference between the strains. The greatest differences between E. coli WT and the Δ*trhA_EC_* strain were for membrane pore-forming antibiotics (gramicidin D) and antibiotics targeting transmembrane ion gradients (nigericin and valinomycin). Membrane potential also affects the ability of cells to form and communicate within and between biofilms (13, 14), and we found that the Δ*trhA_EC_* strain produced roughly 1.5 times as much biofilm as E. coli WT (Fig. 4D). The pleiotropic phenotypes are consistent with TrhA’s role in membrane energetics.

### TrhA homologs in other bacteria function in membrane energetics homeostasis linked to FA metabolism.

Given the conservation of TrhA homologs in several other bacteria, we speculated that their functions should also be conserved. Comparison of the Δψ of a different strain of E. coli, UB1005, and its derivative lacking TrhA (Δ*trhA_UB_*) using ThT and DiOC_2_(3) indicated the Δ*trhA_UB_* mutant had a hyperpolarized Δψ relative to its parent, regardless of the reporter probe used (Fig. S5A and B). The hyperpolarized Δψ of the Δ*trhA_UB_* mutant, opposite to that of the mutant derivative of MG1655, was associated with reduced growth in the presence of acid, alkaline, or salt stresses, while sorbitol had no apparent effect (Fig. S5C to G). Therefore, the deletion of *trhA* in UB1005 and MG1655 yields opposite effects on growth with protons or salt stress. This pattern of behavior is consistent with a dysregulated maintenance of membrane energetics in the mutant lacking TrhA relative to their parent strains and an inability of mutant cells to establish a WT-like membrane potential under given growth conditions. We refer to this mutant phenotype as a dysregulation of membrane potential homeostasis because E. coli strains lacking TrhA experience either a membrane de- or hyper-polarization relative to their parent under various growth conditions. We do not yet know what provokes this opposite effect, but it is possible that the genetic background of UB1005 (harboring additional mutations in *relA* and *spoT* genes [58]) contributes to this difference.

**FIG 8:**
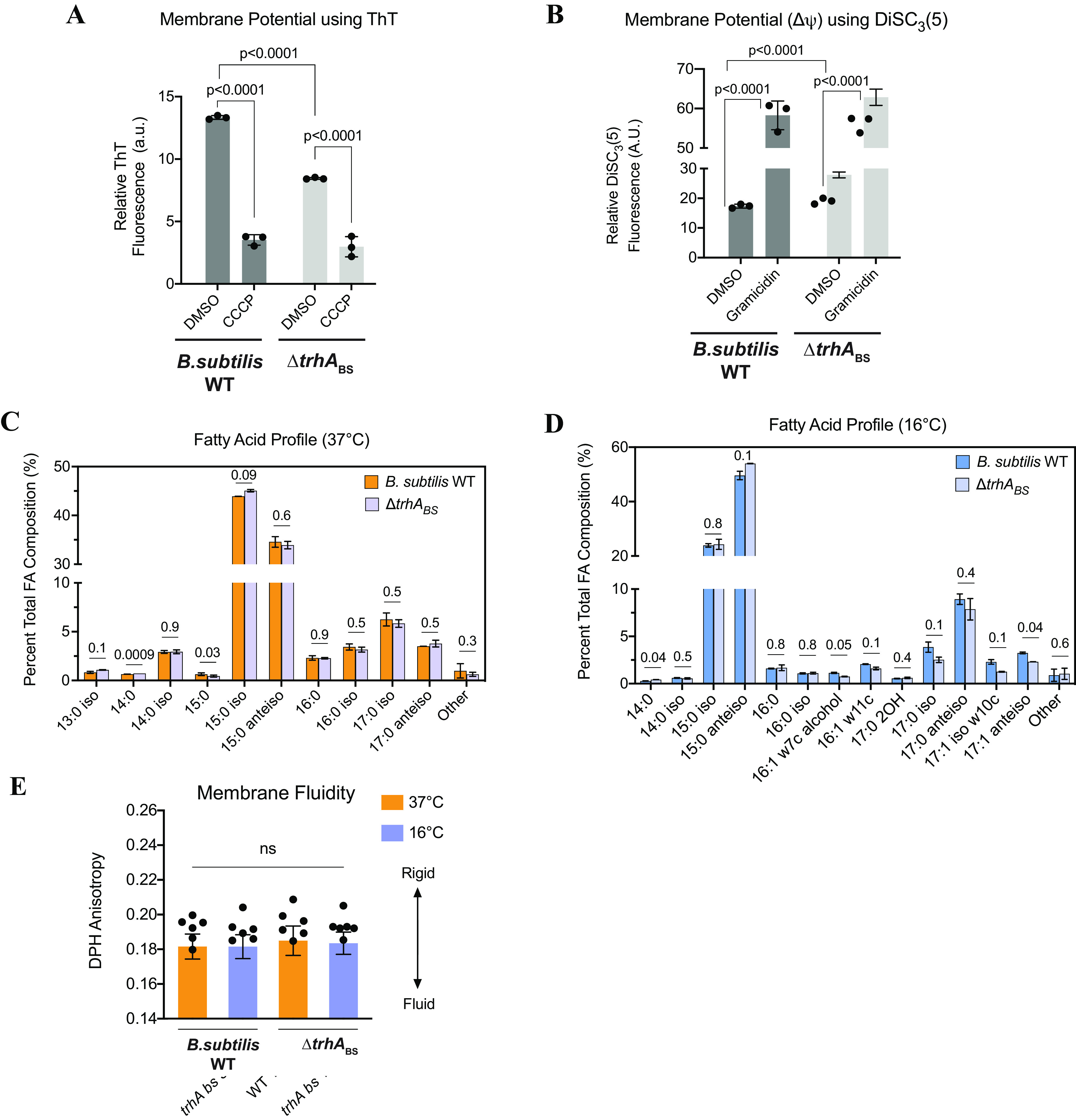
The TrhA homolog in Bacillus subtilis affects membrane potential. (A) Membrane potential measurements of B. subtilis WT and the Δ*trhA*_BS_ strain using ThT reporter. DMSO treatments represent the resting membrane potential of cells. The membrane potential of each strain was collapsed when treated with CCCP, as indicated by a decrease in fluorescence of ThT relative to that of the DMSO control. Data are representative of a single experiment, with one biological replicate and three technical replicates. (B) Membrane potential measurements of B. subtilis WT and the Δ*trhA*_BS_ mutant using DiSC3(5) reporter. Fluorescence of the polar, hydrophobic DiSC3(5) dye is quenched as it enters polarized cells. Because CCCP is incompatible for use with DiSC_3_(5) in this assay (58), we used gramicidin D to depolarize Δψ. Upon membrane depolarization using gramicidin, DiSC3(5) is released back into the free medium, and fluorescence is dequenched. The data are representative of a single experiment, with one biological replicate and three technical replicates. (C) Total fatty acid composition of B. subtilis WT and the Δ*trhA*_BS_ strain grown at 37°C. (D) Total fatty acid composition of B. subtilis WT and the Δ*trhA*_BS_ strain grown at 16°C. Error bars represent the standard deviation of two biological and two technical replicates. *P* values are listed above each set of bars. (E) Membrane fluidities of B. subtilis WT and the Δ*trhA*_BS_ strain grown at 37°C and 16°C. Membrane fluidity of whole cells was measured using DPH anisotropy, where higher values in anisotropy indicate a more rigid membrane. Data represent six biological replicates, where each point is the average of three technical replicates. Error bars represent the standard deviation.

We also characterized the role of the TrhA homolog (encoded by the *yplQ* gene) in the distantly related Bacillus subtilis strain 168 and a mutant derivative lacking the TrhA homolog (Δ*trhA_BS_*). Using two different reporter dyes, previously used to measure Δψ of B. subtilis (59), we showed that the resting Δψ of B. subtilis WT was higher (at least 50% more) than that of the Δ*trhA_BS_* mutant (Fig. 8A and B). The findings indicate that TrhA_BS_ supports its role in membrane potential homeostasis in B. subtilis.

To determine if the lack of TrhA_BS_ also provoked changes in membrane FA composition, we compared the FA composition of the B. subtilis WT and Δ*trhA_B_*_S_ mutant membranes and found subtle differences at 37°C versus 16°C. At 37°C, the only differences were that the Δ*trhA_B_*_S_ mutant produced more C14:0 than B. subtilis WT (Fig. 8C). At 16°C, the Δ*trhA_B_*_S_ strain produced more C14:0 and fewer C17:1 anteisos than B. subtilis WT (Fig. 8D). Despite these changes, the Δ*trhA_B_*_S_ mutant made the same adjustments that B. subtilis WT made to its FA profile when acclimating from 37°C to 16°C (Fig. S6A and B), and net membrane fluidity was unaffected (Fig. 8E). Thus, mutants lacking *trhA* in B. subtilis and E. coli have similar subtle changes in their FA profiles. These changes are small yet consistent across the strains analyzed, suggesting that TrhA has some unknown, small effects on FA metabolism in both E. coli and B. subtilis.

## DISCUSSION

Here, we present evidence that TrhA homologs, which are distantly related to eukaryotic PAQRs, are required for homeostasis of membrane energetics in two E. coli strains and in B. subtilis using defined mutants, physiological assays, and evaluation of membrane potential using fluorescent dye reporters as a read-out of membrane energetics status. The following lines of evidence support this conclusion: the bacterial mutant strains lacking TrhA homologs (i) possess a dysregulated membrane potential relative to the WT in E. coli and B. subtilis under various growth conditions, (ii) display either depolarized or hyperpolarized membrane potential relative to WT, (iii) show pleiotropic physiological changes consistent with dysregulated membrane energetics, and (iv) experience further depolarization or hyperpolarization in response to changes in proton or salt gradients in E. coli. Given the phylogenetic distance of B. subtilis and E. coli, control of membrane energetics homeostasis is the likely shared function of bacterial TrhA homologs.

The membrane proteomes of a Δ*trhA_EC_* strain revealed concurrent physiological stresses and altered cellular metabolism, which suggested altered membrane energetics with dysregulated membrane potential homeostasis in the mutant relative to the WT, confirming these observations. Perturbation of the bacterial membrane potential is associated with drug potency (57), motility defects (60, 61), and an inability to achieve pH homeostasis (20). We observed each of these phenotypes for E. coli lacking TrhA_EC_. Depolarized cells have destabilized membranes, protein mistranslation, and misfolding that is not severe enough to cause cell death (17), consistent with the Δ*trhA_EC_* mutant’s ability to grow similarly to the WT. Proteomics changes in the Δ*trhA_EC_* mutant compared to the WT indicated that the mutant adjusts its metabolism and physiology to grow under laboratory conditions by reorganizing its metabolism to maintain essential transport and metabolic processes, minimizing transmembrane ions and metal fluxes and elevating cellular stress responses to charged molecules.

The cytoplasm of E. coli becomes acidified during acid and osmotic stress, and E. coli uses similar mechanisms to cope with these stressors (62). Altered acid, alkaline, and salt stress responses compared to parent strains were observed in both Δ*trhA_EC_* and Δ*trhA_UB_*. Exposure to acidic pH decreases the PMF of E. coli and depolarizes the Δψ (63). Exposure to external high pH artificially increases the PMF of E. coli WT and hyperpolarizes the Δψ (64). Here, we observed similar Δψ changes for the E. coli MG1655 strain. However, the Δψ of Δ*trhA_EC_* became hyperpolarized under both high and low external pH conditions. The mutant response is consistent with altered membrane potential homeostasis (i.e., WT-like) in Δ*trhA_EC_*. Changes in external pH or salt concentrations led to reduced growth for both WT and mutant pairs. However, under these conditions, growth of Δ*trhA_EC_* was greater than its parent, while growth of Δ*trhA_UB_* was further reduced compared to its parent. These responses tracked the depolarized (Δ*trhA_EC_*) or hyperpolarized (Δ*trhA_UB_*) Δψ of these mutants compared to their parents. We surmise that low or high external pH and high salts conditions artificially further increase (Δ*trhA_EC_*) or decrease (Δ*trhA_UB_*) the ΔpH and/or Δψ of the mutants compared to parent strains. While we do not know how these opposite effects are produced, these responses are consistent with our hypothesis that the mutants have an altered ability to maintain membrane potential homeostasis.

We do not yet know the mechanism(s) by which TrhA modulates membrane potential homeostasis in bacteria. However, the data obtained here, combined with information available from distant eukaryotic homologs, hint at a possible mechanism(s) to be investigated in the future. Eukaryotic class I PAQRs from humans and C. elegans control the abundances of unsaturated FAs in the membrane in response to conditions that decrease membrane fluidity, such as low temperature or dietary fatty acids (27–30, 41–43, 45). Our study demonstrates that the bacterial TrhA (PAQR class III) homologs do *not* primarily control membrane fluidity. However, our data suggest a role for TrhA in FA metabolism. TrhA_EC_ is part of the FabR regulon and is expressed similarly to *fabAB*, implying that TrhA_EC_ is expressed under conditions where long unsaturated FAs are produced. The FA profiles of mutants lacking TrhA in both E. coli and B. subtilis have small but consistent changes relative to parent strains—reduction in abundance of long, unsaturated FAs and accumulation of short, saturated FAs. Furthermore, PAQR family proteins are found in *Bacteria* phyla in which membranes include ester-linked fatty acids, but not in *Archaea* (24) with isoprenoid lipids (65). Membrane lipids are essential to membrane protein folding and function (4). In the absence of a molecular mechanism, we cannot dissociate whether the dysregulated membrane potential which occurs with a lack of TrhA is through changes in membrane protein abundances, suggested by proteomics data for the MG1655 and mutant pair, or whether it is associated with an effect of TrhA on membrane FA composition, which was observed for both E. coli strains and B. subtilis WT and mutant pairs analyzed here. Based on the evidence here, together with the role of distantly related eukaryotic PAQRs known to date and the phylogenetic distribution of TrhA bacterial homologs, we favor the hypothesis that TrhA homologs mediate their effect on membrane potential homeostasis through their effect on unsaturated FA metabolism.

The protein sequence of bacterial TrhA homologs and of the most closely related class III eukaryotic PAQRs are significantly shorter than other eukaryotic PAQR homologs from classes I and II studied to date (24). The longer eukaryotic homologs may thus have evolved divergent functions relative to the ancestral class III homologs, such as binding ligands such as adiponectin or modulating membrane fluidity. The findings here lead us to propose that a common function of PAQR family proteins is homeostasis of the membrane (membrane fluidity or energetics) linked to fatty acid metabolism.

## MATERIALS AND METHODS

### Strains and media.

The bacterial strains used in this study are listed in Table 1. E. coli and B. subtilis strains were grown at 37°C in Luria broth (LB), minimal M9 medium containing glucose or oleate, or on LB agar plates supplemented with antibiotics (100 μg/mL ampicillin, 34 μg/mL chloramphenicol, or 50 μg/mL kanamycin). Inducers were used at 1 mM IPTG (isopropyl β-d-1-thiogalactopyranoside) (pRH005 and pTrc99a) or 1% l-arabinose (pBAD33). The Δ*trhA* strain derivatives of E. coli MG1655 and UB1005 were generated by Lambda-red recombination (66). Bacillus subtilis 168 and the Δ*trhA_BS_* strain were obtained from the *Bacillus* Genetic Stock Center (BGSC).

**TABLE 1:** List of bacterial strains, plasmids, and primers used in this study^*a*^

Strain, plasmid, or primer	Relevant properties	Reference or source
Strains		
Escherichia coli MG1655	*F*-, λ-, *ilvG*-, *rfb-50*, *rph-1*	76
Δ*trhA_EC_*	*F-*, λ*-*, *ilvG-*, *rfb-50*, *rph-1*, *ΔtrhA_EC_*::*Km*	This work
Δ*fadR*	*F-*, λ*-*, *ilvG-*, *rfb-50*, *rph-1*, Δ*fadR*	67
Δ*fadR*Δ*fabR*	P1 transduction of Δ*fabR*::*Km* (Keio collection) in Δ*fadR*. Km cassette removed using pCP20 plasmid.	This work
MG1655 (pRH005)	MG1655 containing empty (pRH005) (Km, Cm)	This work
Δ*trhA_EC_* (pRH005)	Δ*trhA_EC_* containing empty (pRH005) (Km, Cm)	This work
Δ*trhA_EC_* (pRH005 *trhA_EC_*)	Δ*trhA_EC_* containing (pRH005 *trhA_EC_*) (Km, Cm)	This work
MG1655 (pBAD33)	MG1655 containing empty (pBAD33) (Cm)	This work
Δ*trhA_EC_* (pBAD33)	Δ*trhA_EC_* containing empty (pBAD33) (Cm)	This work
Δ*trhA_EC_* (pBAD33 *trhA_EC_*)	Δ*trhA_EC_* containing (pBAD33 *trhA_EC_*) (Cm)	This work
MG1655 (pTrc99a)	MG1655 containing empty (pTrc99a) (Ap)	This work
Δ*trhA_EC_* (pTrc99a)	Δ*trhA_EC_* containing empty (pTrc99a) (Ap)	This work
Δ*trhA_EC_* (pTrc99a *trhA_EC_*)	Δ*trhA_EC_* containing (pTrc99a *trhA_EC_*), (Ap)	This work
Escherichia coli Top10	General cloning strain	Invitrogen
Escherichia coli UB1005	F-, λ-, *gyrA37*(NalR), *relA1*, *spoT1, metB1*, λ^R^	77
UB1005 Δ*trhA_EC_*	F-, λ-, *gyrA37*(NalR), *relA1, spoT1, metB1*, λ^R^, *ΔtrhA::Km*	This work
Bacillus subtilis 168	*trpC2*	78; BGSC
Δ*trhA_BS_*	*trpC2*, *ΔyplQ*::*Erm*	78; BGSC
Plasmids		
pUA66	ori sc101, GFPmut2 (Km)	40
pUA-*fabB*	*fabB* promoter in pUA66	67
pUA-*fabA*	*fabA* promoter in pUA66	67
pUA-*trhA_EC_*	*trhA_EC_* promoter in pUA66	40
pUA-*trhA*_EC_mut	Site-directed PCR mutagenesis in the FabR binding site on the pUA- *trhA_EC_* plasmid	This work
pJL72	TAP tag sequence	68
pUA-TAP	TAP tag sequence from pJL72 (BamHI/EcoRV) in pUA66 (BamHI/HincII)	This work
pUA-TrhA_EC_-TAP	*trhA_EC_* gene (promoter and ORF) cloned in phase with TAP sequence in pEB1588(XhoI/BamHI)	This work
pDONR221	Gateway-based cloning vector (Km)	Invitrogen
pRH005	Gateway-based destination vector expressing proteins fused with YFP at the C terminus; ori RK2*oriV*, (Km, Cm)	51
pRH005 *trhA_EC_*	pRH005 containing WT *trhA_EC_*, (Km, Cm)	This work
pTrc99a	Expression vector with inducible LacI promoter; ori ColE1/pMB1/pBR322, (Ap)	79
pTrc99a *trhA_EC_*	pTrc99a containing *trhA_EC_* (Cm)	This work
pBAD33	Expression vector with arabinose-inducible promoter; ori pACYC184/p15A (Cm)	80
pBAD33 *trhA_EC_*	pBAD33 containing *trhA_EC_* (Cm)	This work
Primers		
*trhA_EC_* Fwd	CACCATGGTTCAGAAGCCCCTC	
*trhA_EC_* Rev	TTACGCCTGCCCAATATA	
UP *trhA_EC_* XhoI	ttgctcgagGAAAATATGACCCTGACTGAACTG	
*trhA_EC_* BamHI Rev	cgggatccCGCCTGCCCAATATACAAATAGATC	
*trhA_EC_*mut FabR site Fwd	CAGAATTTATTTTAGCTAGTGGGTGTTCACTGGAAC	
*trhA_EC_*mut FabR site Rev	GTTCCAGTGAACACCCACTAGCTAAAATAAATTCTG	
GW *trhA_EC_* Fwd	GGGGACAAGTTTGTACAAAAAAGCAGGCTATGGTTCAGAAGCCCCTCATT	
GW *trhA_EC_* Rev	GGGGACCACTTTGTACAAGAAAGCTGGGTCCGCCTGCCCAATATACAA	
GW *pgk* Fwd	GGGGACAAGTTTGTACAAAAAAGCAGGCTATGTCTGTAATTAAGATGACC	
GW *pgk* Rev	GGGGACCACTTTGTACAAGAAAGCTGGGTCCTTCTTAGCGC GCTCTTC	
pRH005 *ccdB* mut Fwd	ATGTTCTAAAGCAGGTAAATGTCAGGC	
pRH005 *ccdB* mut Rev	GCCTGACATTTACCTGCTTTAGAACAT	
XbaI *trhA_EC_* Fwd	TAAGCATCTAGATCTAGGAGGTAAGTTATGGTT	
HindIII *trhA_EC_* Rev	TGCTTAAAGCTTTTACGCCTGCCCAATATA	

a Km, kanamycin; Cm, chloramphenicol; Ap, ampicillin; Erm, erythromycin; BGSC, *Bacillus* Genetic Stock Center. The restriction enzymes used are underlined as part of the primer name, and the location of these restriction sites within the primer is underlined.

### Plasmid construction and genome sequencing.

The pUA66, pUA-*trhA_EC_*, pUA-*fabA*, and pUA-*fabB* plasmids were obtained from the library of E. coli promoters fused to GFP coding sequence (39) or constructed previously (66). PCR site-directed mutagenesis using primers *trhA_EC_* mut FabR site forward (Fwd) and *trhA_EC_* mut FabR site reverse (Rev) (Table 1) was used to mutate the FabR binding box in the *trhA_EC_* promoter. Plasmid pUA66 was modified for translational fusion by introducing the TAP tag sequence from plasmid pJL72 (67). The sequence of *trhA_EC_* (natural promoter and coding sequence) was introduced using the XhoI and BamHI sites of pUA66. Gateway technology, as per the manufacturer’s protocol (Invitrogen), was used to clone into pRH005 (51) (Table 1). Cloning in the pTrc99a and pBAD33 plasmids (Table 1) was completed by restriction digestions and ligation using primers XbaI *trhA_EC_* Fwd and HindIII *trhA_EC_* Rev (Table 1). All plasmids were sequence-verified before being introduced into E. coli by transformation. The genomes of the E. coli MG1655 and Δ*trhA_EC_* strains were sequenced at the Microbial Genome Sequencing Center (MiGS; Pittsburgh, PA, USA).

### Transcriptional fusion analysis.

Transcriptional GFP fusions in the Δ*fadR* and Δ*fadR*Δ*fabR* E. coli strains were done as described in reference 67.

### SDS-PAGE and Western blotting.

Total cell extracts were prepared by resuspending cell pellets in Laemmli buffer 1× at a concentration of 0.3 optical density at 600 nm (OD_600_) in 10 μL (we did not heat, as we observed that this prevents correct migration/detection of the integral TrhA protein in SDS-PAGE), followed by electrotransfer onto nitrocellulose membranes. TrhA-TAP-tagged protein was detected with peroxidase-antiperoxidase antibodies (Sigma) and colorimetric, detection with diaminobenzidine.

### Fatty acid methyl ester (FAME) analysis.

Whole-cell FAME analysis of E. coli and B. subtilis was done by gas chromatography-mass spectrometry (GC-MS) through Microbial ID (Newark, DE) using cultures grown at 37°C or 16°C to early stationary phases (OD_600_ of 1.5 to 2.0).

### DPH anisotropy.

The fluorescent dye DPH (1,6-diphenyl-1,3,5-hexatriene) was used to measure anisotropy, as described previously (46).

### Membrane Proteomics.

Membrane protein fractions were prepared as described in reference 68. Bacterial cell membrane proteins were denatured, reduced, alkylated, and enzymatically digested into peptides using previously established methods (69, 70). All samples were analyzed on a Q Exactive Plus mass spectrometer (Thermo Fisher Scientific) coupled with a Proxeon EASY-nLC 1200 liquid chromatography (LC) pump (Thermo Fisher Scientific). Peptides were separated on a 75-μm inner diameter microcapillary column packed with 30 cm of Kinetex C_18_ resin (1.7 μm, 100 Å; Phenomenex) that was heated to 60°C in a Phoenix S&T NanoLC column heater. For each sample, 2- μg aliquots of peptides were loaded in buffer A (0.1% formic acid, 2% acetonitrile) and eluted with a linear 210-min organic gradient followed by a wash and column reequilibration as follows: 0% to 2% solvent B over 27 min, 2% to 25% solvent B over 148 min, 25% to 50% solvent B over 10 min, 50% to 0% solvent B over 10 min, hold at 0% solvent B for 15 min. The flow rate was kept at 200 nL/min. MS data were acquired with Thermo Xcalibur software v4.27.19 using the topN method, where N can be up to 10.

Data were processed in Proteome Discoverer v2.3 (Thermo Scientific) using MS Amanda v2.0 (71) and Percolator (72). Spectral data were searched against the E. coli reference proteome database, and common laboratory contaminants and identifications were controlled at a false-discovery rate (FDR) of <1% at the peptide level. Proteins were quantified according to the sum of their peptide chromatographic areas under the curve and normalized in Proteome Discoverer by the total peptide amount. To identify differential protein abundances, hypothesis testing was performed by *t* tests, and adjusted *P* values were calculated based on the background population of proteins. Log_2_ protein abundance differences were determined to assess the fold change. Proteins were assigned to appropriate Gene Ontology (GO) term categories using EcoCyc. These data are available in the ProteomeXchange Consortium via the MASSIVE repository (https://massive.ucsd.edu/; username, MSV000088025_reviewer; password, ecoli_membrane).

### Measurement of Δψ using DiOC_2_(3), ThT, and DiSC_3_(5).

Colonies of E. coli and B. subtilis were grown overnight at 37°C in LB broth, reinoculated (250 μL into 3 mL of fresh LB), and shaken for 2.5 h at 37°C. Membrane potential was measured as described (59, 73).

### Physiological assays.

Growth of E. coli was determined in 200 μL of LB in a 96-well plate with shaking using an ELx808 absorbance microplate reader (Biotek). The specific growth rate, μ, was calculated as described (74). The ATP concentration was quantified using the BacTiter-Glo kit luminescence assay as per the manufacturer’s protocol (Promega). Biofilms were quantified as described (75).

For soft agar assays, five5 μL of culture was inoculated into the center of plates containing terrific broth (TB) (12 g/L tryptone, 10 g/L yeast extract, 0.4% glycerol [vol/vol]), 10% 1× phosphate-buffered saline [PBS]) with 0.3% agar (wt/vol). Plates were incubated for 15 h at 28°C.

For acid, alkaline, and osmotic stress tests, the pH of Luria Broth (LB) was adjusted to 4.5, 7.0, or 9.0 using HCl or NaOH or supplemented with 0.75 and 1 M for NaCl, 1.0 M and 1.5 M Sorbitol, and 0.5 M and 0.75 M KCl. Growth in stress conditions was determined as described above.

For MICs, cells were grown in LB to the log phase, diluted in 200 μL of fresh LB containing antibiotics at concentrations denoted in Fig. 4C, and shaken overnight using an ELx808 absorbance microplate reader (Biotek). Concentration (MIC) values were determined by the lowest antibiotic concentration added to the medium at which the growth rate was inhibited.

### Statistical analyses.

Statistical analyses were performed in GraphPad Prism 9. Two-tailed *t* tests were applied for normally distributed data with one degree of freedom. Exact *n* values are provided in each figure. Representative images or results are shown for soft agar motility assays and DiSC_3_(5), where experiments were repeated at least three times.
