# Supplementary material for: Bacterial Homologs of Progestin and AdipoQ Receptors (PAQRs) Affect Membrane Energetics Homeostasis but Not Fluidity
Source: J Bacteriol. 2022 Mar 14;204(4):e00583-21. doi: 10.1128/jb.00583-21 (PMC9017321; doi:10.1128/jb.00583-21)
Supplement: SUPPLEMENTAL FILE 1 — Tables S1 to S4; Fig S1 to S6. Download jb.00583-21-s0001.pdf, PDF file, 7.0 MB [file jb.00583-21-s0001.pdf]

Table S1A. Proteins found in decreased abundance in ΔtrhAEC vs. E. coli WT.

GO-TERM	Δ*trhA*$_{EC}$ / *E. coli* WT 37°C DOWN	Δ*trhA*$_{EC}$ / *E. coli* WT 16°C DOWN
FLAGELLA & CHEMOTAXIS	CheY, FliJ, FlgD, YjcZ	FliDEIMP, FlgA
TRANSCRIPTIONAL REGULATION	----------	FlhD, Fnr, TdcA
RESPIRATION & OXIDATION-REDUCTION	CyoA, FrdD, NarGH, NuoJ, SdhD, YdhV, YdiJ, YnfE	HybA, NapC, NuoB
MEMBRANE MODIFICATION		
Peptidoglycan Synthesis	EnvC, MltBC, MrcB	----------
LPS Synthesis	LptDE, RfaP, WbbI	----------
Lipid & Fatty Acid Metabolism	FabAF, LpxDK, PldA, RssA	----------
Lipoprotein & Lyso-phospholipid Transport	LolBE, LplT	----------
TRANSPORTERS	HisQ, ModC, TolC, YjcE	LamB
Metal Transporters	FeoB, FhuA, NikC, ZntA	----------
Proton Symporters	BtuB, DctA	BtsT, NanT, TnaB
Proton Antiporters	DcuC, NepI	----------
PTS Systems	----------	SrlAE
OMPS, PROTEIN COMPLEXES, & LIPOPROTEINS	AsmA, AtpDHG, BamABD, LepB, RcsF, TamA, YecR	OmpWX
DNA REPLICATION & TRANSCRIPTON		
DNA Replication	----------	DnaQ

tRNA Modification	----------	TtcA
Transcription	RpoC	----------
Ribosome Assembly	----------	DbpA
STRESS RESPONSE & PROTEIN REPAIR	YbfA	HtpG, UspE, Ves
METABOLISM		
Amino Acid Metabolism	AnsB, GcvP	AroE, AspA
Carbon Metabolism	NanA	GlcB
Cofactor Metabolism	MoaB	----------
Nucleotide Metabolism	PurL	----------
OTHER		
Cell Division	EngB	----------
Protein Maturation	HypD	----------
Signal Transduction	DcuS	GlrK, YrfF
Protease	SohB, SppA	----------
UNKNOWN	YcjF, YdjY, YtfL, YtjB	----------

Table S1B. Proteins found in increased abundance in Δ*trhA*$_{EC}$ vs. *E. coli* WT.

GO-TERM	Δ*trhA*$_{EC}$ / *E. coli* WT 37°C UP	Δ*trhA*$_{EC}$ / *E. coli* WT 16°C UP
TRANSCRIPTIONAL REGULATION	BolA, MprA, NrdR, OmpR, SlyA, StpA, PdhR, UxuR, YeeN	GadW
RESPIRATION & OXIDATION-REDUCTION	GhrAB, GrxBD, HcxB, MsrC, NarY, YgiN, YjhC	c, YeaE
MEMBRANE MODIFICATION		
Peptidoglycan Synthesis	MurB, YbiS, YnhG	----------
LPS Synthesis	GalF, GlmU, HldD	----------
Lipid & Fatty Acid Metabolism	FabG, TesB, YbfF	FabH
TRANSPORTERS	----------	YbbA
Proton Symporters & Antiporters	----------	MntH
PTS Systems	AgaD, GatA	----------
DNA REPLICATION, TRANSCRIPTION, & TRANSLATION		
DNA Replication, Modification, and Repair	DnaB, ExoX, HsdR	----------
tRNA Modification	MiaB, SelD	MnmE, RlmJ, RsmH, YcaO
Transcription	CsiE, GreA, NusG	----------
Translation	PrfA, RplM, RpsF, TyrS	----------
PTM	PrmA, RimI, RoxA	----------
RNA Processing	RbfA, Rnc, RsmG, RlmH	----------

STRESS RESPONSE & PROTEIN REPAIR	Bcp, ClpB, DegQ, DinD, GadA, GloA, GstB, Pcm, TreA, YgfA, YtfE	HslV
METABOLISM		
Amino Acid Metabolism	AroK, AsnAB, MetH, ThrBC, TyrA	ArgG, IlvL
Carbon Metabolism	AceAF, FumD, GalE, GhrAB, Glk, GntK, PfkB, SrlD, UxuA	DhaL, Eda
Cofactor Metabolism	CoaA, HemHL, IspAE, LipA, MoeA, NadE, PanC, RibC, ThiL, UbiC, YajO, YbjI	EntC, IspB, PncB
Nucleotide Metabolism	Adk, Cmk, NrdE, Tmk, SurE, Yjjx	----------
OTHER		
Quorum Sensing	LuxS	----------
Cell Division	ZapA	----------
SAM Biosynthesis & Metabolism	TehB	MetK
Sulfur Metabolism	CysHQ, SseB	----------
Kinase & Phosphatase	YidA, YniA,	----------
Protein Maturation & PTMs	SixA	EpmB
Signal Transduction	----------	PhoU
UNKNOWN	ElaA, Mrp, QueE, YbhK, YbiT, YceH, YcfP, YecA, YeaH, YebC, YfgD, YfhM, YhgF, YihX, YjiM, YjjA, YjjX, YpfH, YqeC, YqjE	MsyB, YacC, YbgA, YbiU, YfhM, YgjP, YnfT

Table S2. Proteins found in decreased or increased abundance in Δ*trhA*$_{EC}$ at both 37°C and 16°C.

GO-TERM	Protein(s)
Proteins found in decreased abundance in ΔtrhA$_{EC}$ relative to E. coli WT	
Flagella, Motility, & Chemotaxis	Aer, CheABRW, FlgBCEFGHIKL, FlhAB, FliCFGHLNOSZ, MotAB, Tap, Tar, Trg, Tsr, PdeH, YcgR
Transporters	Tsx
Proton Symporter	ZntB
OMPs & Lipoproteins	YajG, YncD
Proteins found in increased abundance in ΔtrhA$_{EC}$ relative to E. coli WT	
Transcriptional Regulators	EvgA, NsrR
Nucleotide Metabolism	
RNA Processing	RlmH
Ribosome Assembly	YceD
Redox (Fe-S)	PflA
Amino Acid Metabolism	PxpA
Cofactor Metabolism	IspH
LPS Synthesis	RfbB
Stress Response	RfbB

Table S3. Specific growth rate (*μ*) of *E. coli* WT and Δ*trhA$_{EC}$* grown at 37°C, 28°C, and 16°C.

Strain	37°C	28°C	16°C
E. coli WT	0.48 h^{-1}	0.27 h^{-1}	0.08 h^{-1}
Δ*trhA$_{EC}$*	0.49 h^{-1}	0.25 h^{-1}	0.08 h^{-1}

Table S4. Genotypic changes in Δ*trhA$_{EC}$* relative to *E. coli* WT.

Predicted Mutations

Gene	Annotation	Description	Mutation	Position
insB-5-insA-5	Insertion element IS1	insB-5, insA-5	Δ776 bp	1,978,503
pgk	T151A (ACT→GCT)	phosphoglycerate kinase	T → C	3,072,172
rmuC	L384F (TTG→TTT)	RmuC, putative recombination limiting protein	G → T	4,018,484

Unassigned Missing Coverage

Gene	Size	Description	Start	End
[yqfA]	666 nt	*[yqfA]*	3,042,510	3,043,175

Unassigned New Junction

Gene	Annotation	Description	Position
stfE	pseudogene (37/537 nt)	e14 prophage; putative side tail fiber protein fragment	1,209,619

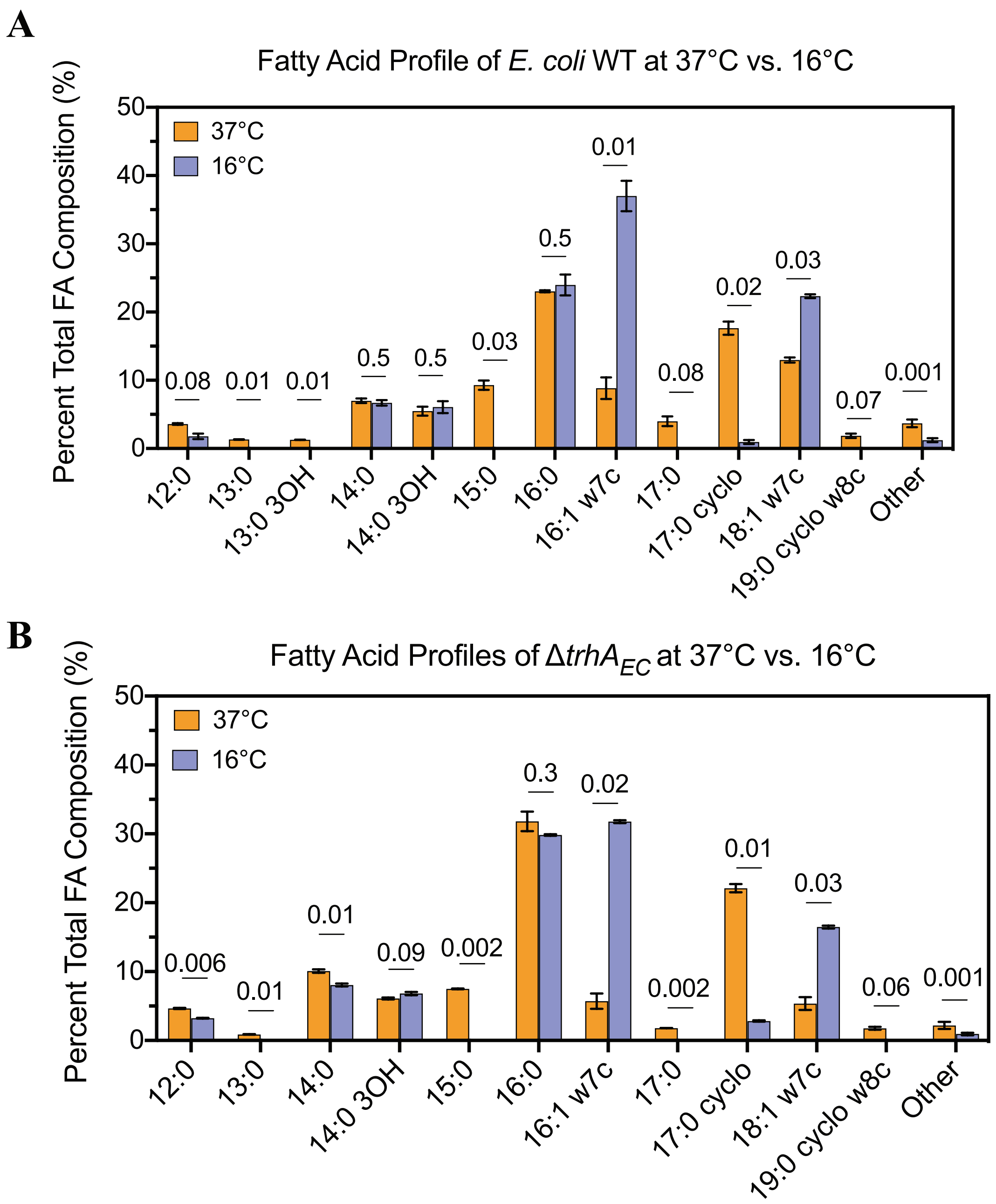

Fig. S1: *E. coli* WT and the Δ*trhA*$_{EC}$ mutant adjust their fatty acid profiles similarly when grown at 37°C vs. 16°C. (A) Total fatty acid composition of *E. coli* WT grown at 37°C and 16°C. (B) Total fatty acid composition of the Δ*trhA*$_{EC}$ mutant grown at 37°C and 16°C. Error bars represent standard deviation of two biological and two technical replicates. P-values are listed above each set of bars.

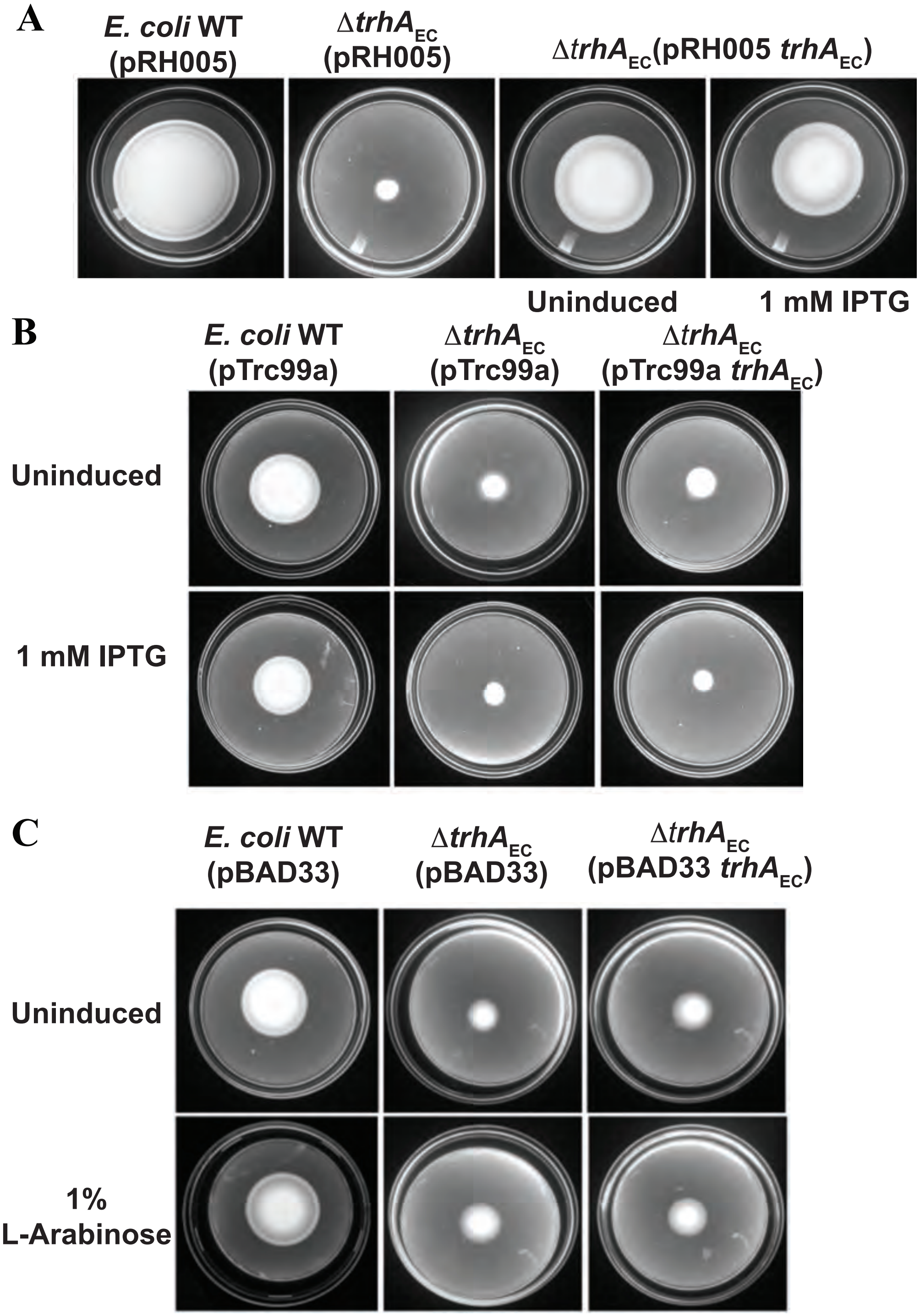

Fig. S2: Complementation of the Δ*trhA*$_{EC}$ mutant works using the pRH005 expression system and does not work using pBAD33 and pTrc99a expression systems. *E. coli* WT and mutant Δ*trhA*$_{EC}$ cells expressing wildtype *trhA*$_{EC}$ from (A) pRH005, (B) pTrc99a, or (C) pBAD33 plasmids were inoculated onto 0.3% soft agar plates. IPTG (1 mM) or L-arabinose (1%) was added to the plates for induction conditions. Plates were incubated at 28°C and imaged after 15 hours.

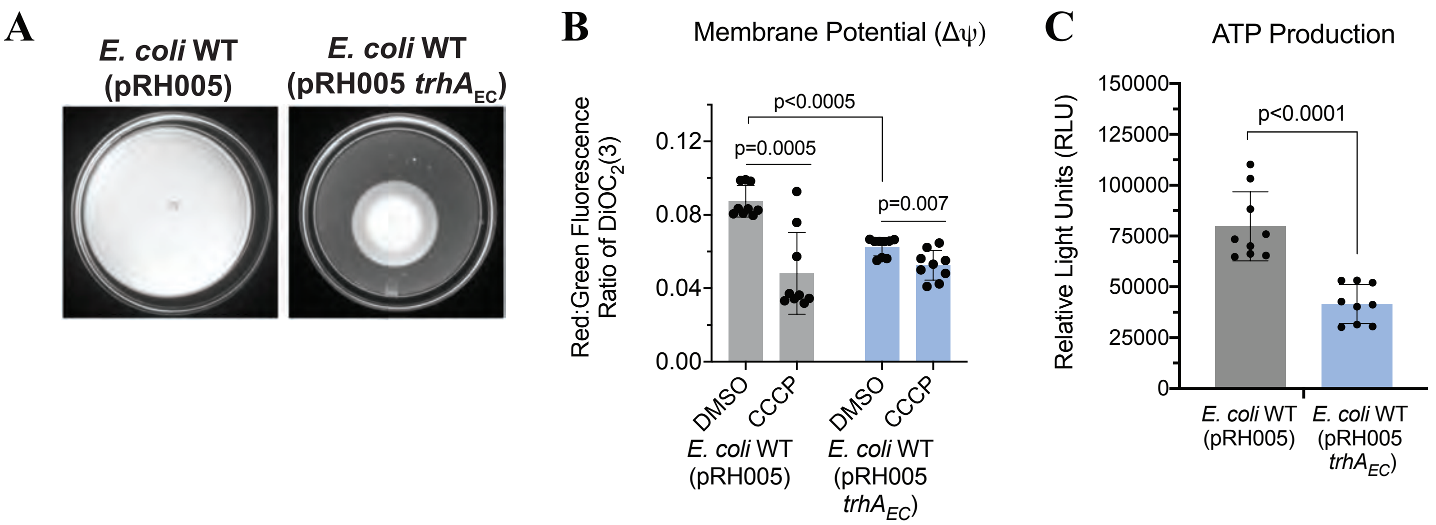

Fig S3: Overexpression of *trh*A$_{EC}$ in the *E. coli* WT background produces a dominant negative phenotype. (A) *E. coli* WT cells overexpressing *trhA*$_{EC}$ were inoculated onto 0.3% agar plates. Plates were incubated at 28°C and imaged after 15 hours. (B) Membrane potential of *E. coli* WT cells over-expressing *trhA*$_{EC}$ was measured using the DiOC2(3) reporter. DMSO treatments represent the resting membrane potential of cells. The membrane potential of each strain was collapsed when treated with CCCP, as indicated by a decrease in the red:green fluorescence ratio (620 nm / 520 nm) of DiOC2(3) relative to the DMSO control. Data represent fluorescence from three biological replicates with three technical replicates. Error bars represent standard deviation. (C) Total ATP production stationary phase *E. coli* WT cells overexpressing *trhA*$_{EC}$ and the empty vector control was measured as luminescence relative light units (RLU). Data represent the ATP content from three biological with three technical replicates. Error bars represent standard deviation.

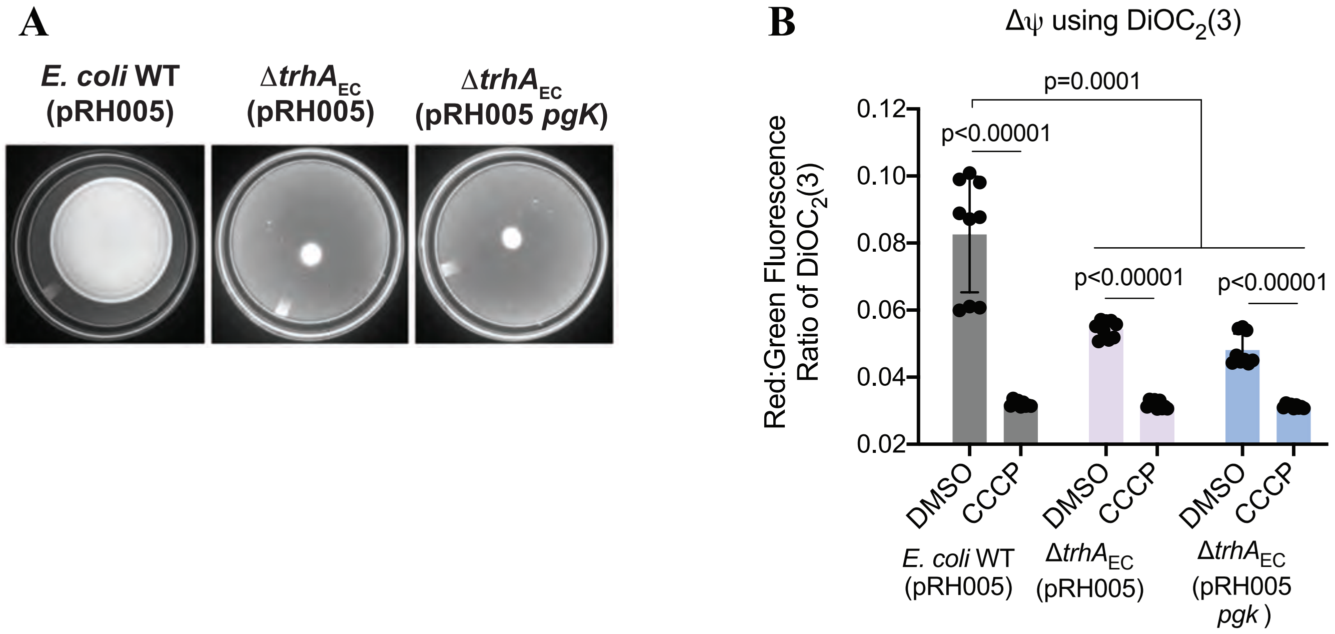

Fig. S4: Secondary mutation in *pgk* gene of the Δ*trhA*$_{EC}$ parental mutant is not responsible for membrane potential-related phenotypes. (A) *E. coli* WT (pRH005), Δ*trhA*$_{EC}$ (pRH005), andΔ*trhA*$_{EC}$ (pRH005 pgk) were inoculated onto 0.3% soft agar plates. Plates were incubated at 28°C and imaged after 15 hours. (B) Membrane potential of *E. coli* WT (pRH005), Δ*trhA*$_{EC}$ (pRH005), and Δ*trhA*$_{EC}$ (pRH005 pgk) was measured using DiOC2(3) reporter. DMSO treatments represent the resting membrane potential of cells. The membrane potential of each strain was collapsed when treated with CCCP, as indicated by a decrease in the red:green fluorescence ratio (620 nm / 520 nm) of DiOC$_2$(3) relative to the DMSO control. Data represent fluorescence from three biological replicates with three technical replicates. Error bars represent standard deviation.

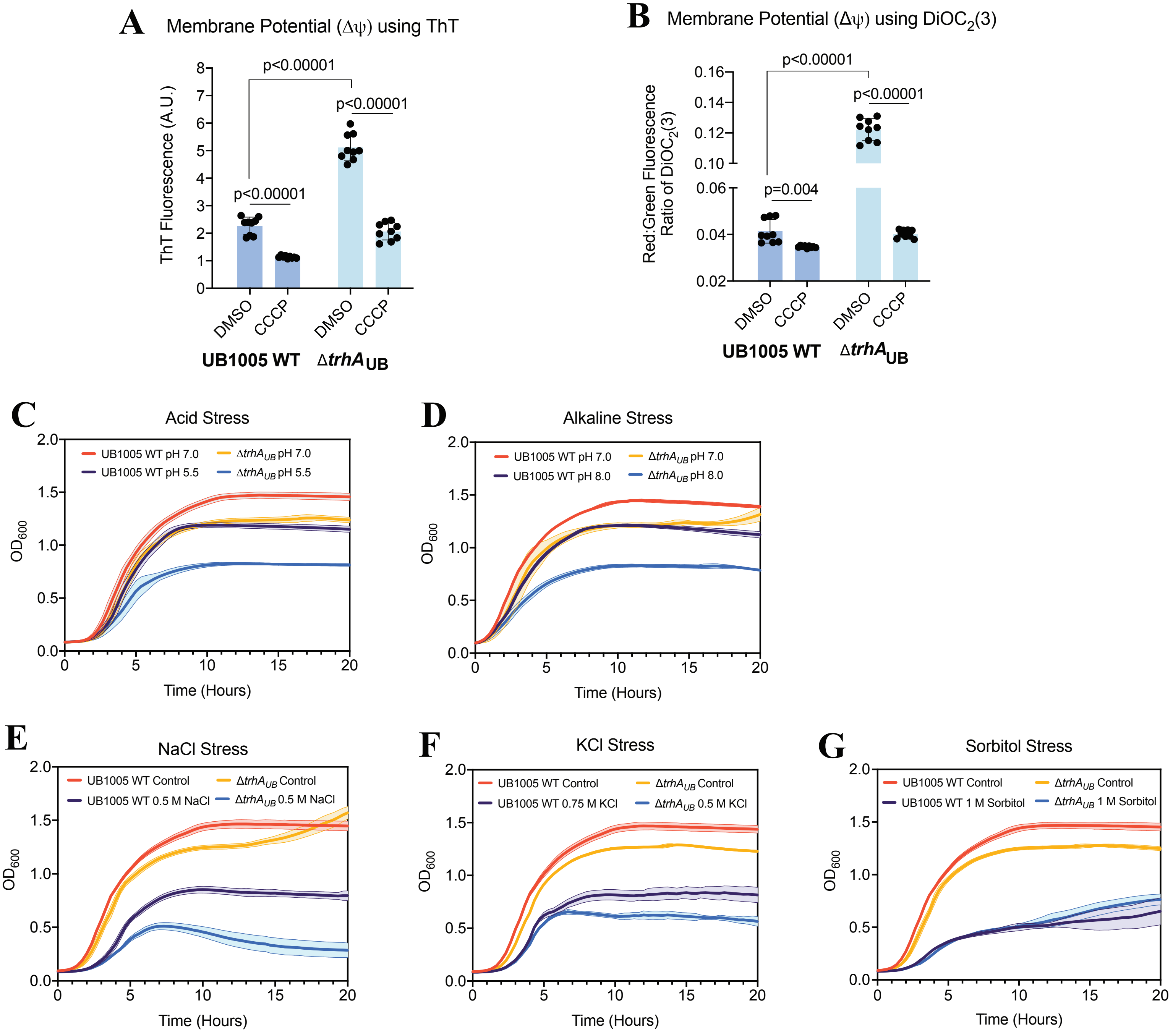

Fig. S5: TrhA homolog in *E. coli* UB1005 affects membrane potential, protons and salt stresses. (A) ThT and (B) DiOC2(3) fluorescent reporters. DMSO treatments represent the resting membrane potential of cells. The membrane potential of each strain was collapsed when treated with CCCP, as indicated by decreased reporter fluorescence. Data represent fluorescence from three biological replicates with three technical replicates. Error bars represent standard deviation. E. coli UB1005 WT and the ΔtrhAUB mutant cells were grown to logarithmic phase at 37°C in LB media at pH 7.0 (C-G) and then shifted to (C) acid stress at pH 5.5, (D) alkaline stress at pH 8.0, (E) NaCl stress at 0.5 M NaCl, (F) KCl stress at 0.5 M KCl, or (G) Sorbitol stress at 1 M sorbitol. All growth curves are representative of the average and standard deviation of three biological with three technical replicates.

A

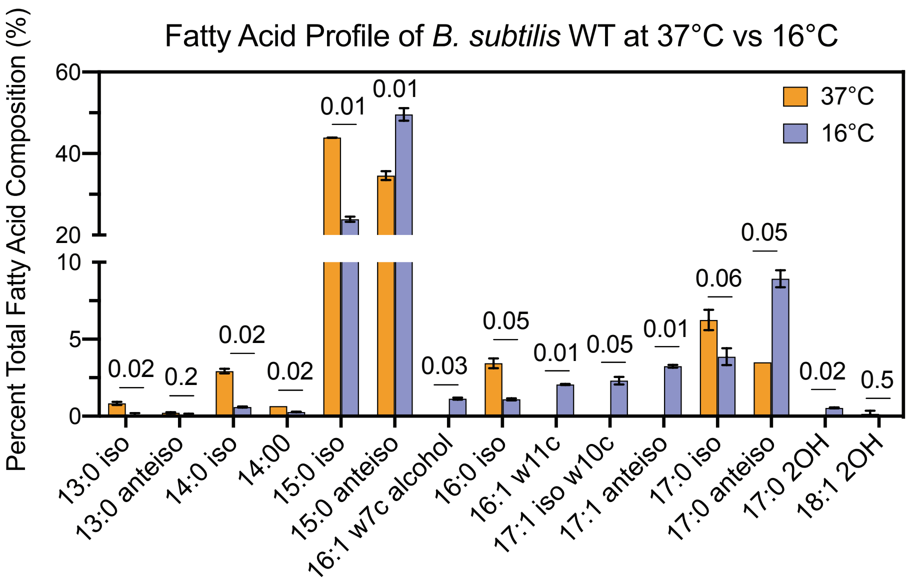

B

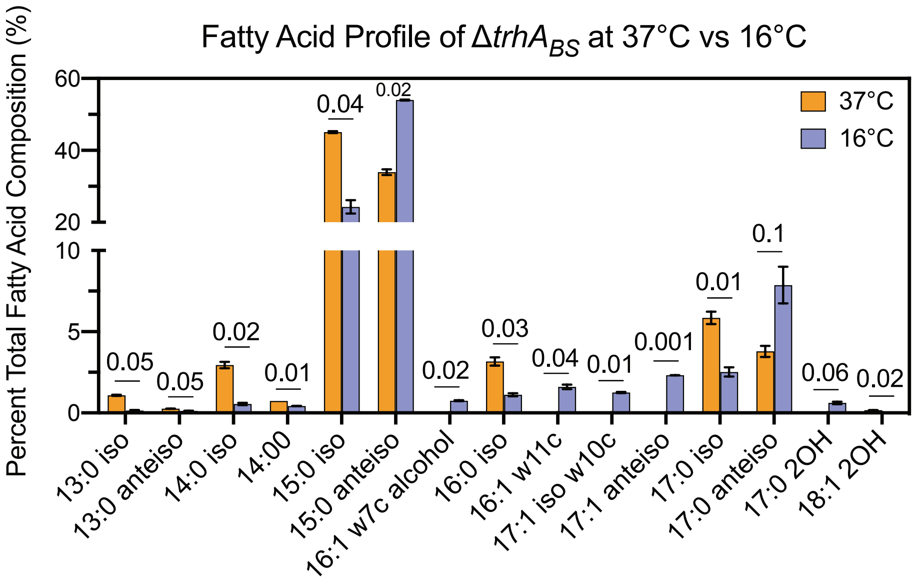

Fig. S6: *B. subtilis* WT and the Δ*trhA*$_{BS}$ mutant adjust their fatty acid profiles similarly when grown at 37°C vs. 16°C. (A) Total fatty acid composition of *B. subtilis* WT grown at 37°C and 16°C. (B) Total fatty acid composition of the Δ*trhA*$_{BS}$ grown at 37°C and 16°C. Error bars represent standard deviation of two biological and two technical replicates. P-values are listed above each set of bars.
